# Probiotic With or Without Fiber Controls Body Fat Mass, Associated With Serum Zonulin, in Overweight and Obese Adults—Randomized Controlled Trial^☆^^☆☆^

**DOI:** 10.1016/j.ebiom.2016.10.036

**Published:** 2016-10-26

**Authors:** Lotta K. Stenman, Markus J. Lehtinen, Nils Meland, Jeffrey E. Christensen, Nicolas Yeung, Markku T. Saarinen, Michael Courtney, Rémy Burcelin, Marja-Leena Lähdeaho, Jüri Linros, Dan Apter, Mika Scheinin, Hilde Kloster Smerud, Aila Rissanen, Sampo Lahtinen

**Affiliations:** a Global Health and Nutrition Science, DuPont Nutrition & Health, FI-02460 Kantvik, Finland; b Smerud Medical Research, N-0212 Oslo, Norway; c Vaiomer S.A.S, FR-31670 Labège, France; d FinnMedi, FI-33520 Tampere, Finland; e Kerava Health Centre, FI-04200 Kerava, Finland; f VL-Medi, FI-00101 Helsinki, Finland; g Clinical Research Services Turku, FI-20520 Turku, Finland; h Obesity Research Unit, University of Helsinki, FI-00290 Helsinki, Finland

**Keywords:** Clinical trial, Fiber, Obesity, Prebiotic, Probiotic, Synbiotic

## Abstract

**Background:**

The gut microbiota is interlinked with obesity, but direct evidence of effects of its modulation on body fat mass is still scarce. We investigated the possible effects of *Bifidobacterium animalisssp. lactis* 420 (B420) and the dietary fiber Litesse® Ultra polydextrose (LU) on body fat mass and other obesity-related parameters.

**Methods:**

225 healthy volunteers (healthy, BMI 28–34.9) were randomized into four groups (1:1:1:1), using a computer-generated sequence, for 6 months of double-blind, parallel treatment: 1) Placebo, microcrystalline cellulose, 12 g/d; 2) LU, 12 g/d; 3) B420, 10^10^ CFU/d in microcrystalline cellulose, 12 g/d; 4) LU + B420, 12 g + 10^10^ CFU/d. Body composition was monitored with dual-energy X-ray absorptiometry, and the primary outcome was relative change in body fat mass, comparing treatment groups to Placebo. Other outcomes included anthropometric measurements, food intake and blood and fecal biomarkers. The study was registered in Clinicaltrials.gov (NCT01978691).

**Findings:**

There were marked differences in the results of the Intention-To-Treat (ITT; *n* = 209) and Per Protocol (PP; *n* = 134) study populations. The PP analysis included only those participants who completed the intervention with > 80% product compliance and no antibiotic use. In addition, three participants were excluded from DXA analyses for PP due to a long delay between the end of intervention and the last DXA measurement. There were no significant differences between groups in body fat mass in the ITT population. However, LU + B420 and B420 seemed to improve weight management in the PP population. For relative change in body fat mass, LU + B420 showed a − 4.5% (− 1.4 kg, *P* = 0.02, *N* = 37) difference to the Placebo group, whereas LU (+ 0.3%, *P* = 1.00, *N* = 35) and B420 (− 3.0%, *P* = 0.28, *N* = 24) alone had no effect (overall ANOVA *P* = 0.095, Placebo *N* = 35). A post-hoc factorial analysis was significant for B420 (− 4.0%, *P* = 0.002 vs. Placebo). Changes in fat mass were most pronounced in the abdominal region, and were reflected by similar changes in waist circumference. B420 and LU + B420 also significantly reduced energy intake compared to Placebo. Changes in blood zonulin levels and hsCRP were associated with corresponding changes in trunk fat mass in the LU + B420 group and in the overall population. There were no differences between groups in the incidence of adverse events.

**Discussion:**

This clinical trial demonstrates that a probiotic product with or without dietary fiber controls body fat mass. B420 and LU + B420 also reduced waist circumference and food intake, whereas LU alone had no effect on the measured outcomes.

## Highlights

• The probiotic B420 and synbiotic LU + B420 seem to control body fat mass in humans, especially in the abdominal area.
• B420 and LU + B420 reduce waist circumference and energy intake, but only LU + B420 increases lean body mass.
• Reduced body fat is associated with zonulin, a marker of epithelial permeability, supporting earlier preclinical findings.

Concise results on probiotics for weight management are lacking. Stenman et al. show in a gold standard clinical study that the probiotic B420 with or without a fiber controls body fat, waist circumference and energy intake when taken in a dietary intervention of six months. Study participants maintained habitual diet and physical activity. Stenman et al. link the benefit to changes in serum zonulin, a potential gut permeability marker. The authors have previously shown in animal studies that these gut microbiota targeting products improve metabolic health in mice by strengthening gut barrier function. These effects are now translated to humans.

## 1 Introduction

The gut microbiota is associated with metabolic disorders such as obesity and type 2 diabetes. Since the discovery of a link between gut microbiota and obesity in 2006 (Turnbaugh et al., 2006, Ley et al., 2006), increasing evidence has been presented to suggest a causal relationship between gut microbiota and metabolic disorders (Stenman et al., 2015a).

One of the mechanisms postulated to explain this relationship is the metabolic endotoxemia hypothesis, which links gut microbes to low-grade inflammation and further to metabolic disorders (Burcelin et al., 2009, Cani et al., 2007). In experimental animals (Stenman et al., 2012, Brun et al., 2007) and also in humans (Teixeira et al., 2012, Leber et al., 2012), obesity and metabolic disorders have been associated with an impaired gut barrier, which may lead to increased translocation of endotoxins (Kallio et al., 2015, Stenman et al., 2014), especially in connection with a Western diet (Pendyala et al., 2012). These highly inflammatory components can ultimately lead to tissue inflammation and, consequently, metabolic disorders (Burcelin et al., 2009).

Dietary recommendations have been put forward to try to prevent metabolic disorders. In the Finnish Diabetes Prevention Study, carried out in a large cohort of middle-aged participants with impaired glucose tolerance, a simple lifestyle intervention reduced the risk of diabetes by 58% compared to control (Tuomilehto et al., 2001). Of those subjects with excellent adherence to the intervention program, meeting four of the five intervention goals, none developed type 2 diabetes. Still, in the general population, the prevalence of metabolic disorders is rapidly increasing, and supportive prevention options—such as those targeting the gut microbiota—need to be developed to support current standards of care.

Probiotics are live micro-organisms that confer health benefits to the host (Hill et al., 2014), whereas prebiotics are fibers that selectively improve the growth of beneficial gut microbes (Gibson and Roberfroid, 1995). In experimental animals, we have previously shown that *Bifidobacterium animalis* ssp. *lactis* 420 (B420) prevented weight gain, improved insulin sensitivity, as well as reduced endotoxemia and tissue inflammation (Amar et al., 2011, Stenman et al., 2014, Stenman et al., 2015b, Garidou et al., 2015). The probiotic B420 has been given to humans in earlier clinical trials, albeit at quite low doses (Klein et al., 2008, Roessler et al., 2008, Kok et al., 1996), but its potential *anti*-obesity effects have remained unexplored. The prebiotic employed in the current study, Litesse® Ultra polydextrose (LU), is a randomly cross-linked polymer of glucose, which remains undigested by the host and may increase the number of *Bifidobacteria* in a colonic continuous culture system (Probert et al., 2004). Its administration has been reported to induce satiation (Ibarra et al., 2015) and to ameliorate the glycemic response to a glucose load (Jie et al., 2000), indicating potential benefits for weight maintenance and metabolic health.

To date, there is no conclusive evidence for the ability of probiotics to control body fat mass in humans. Despite a fair amount of promising clinical findings (Sanchez et al., 2014, Kadooka et al., 2010, Kadooka et al., 2013, Osterberg et al., 2015), randomized controlled trials demonstrating beneficial probiotic effects in the primary statistical analysis of a well-powered study conducted according to Good Clinical Practice (GCP) are lacking. Only few studies on prebiotics have shown effects on weight management (Parnell and Reimer, 2009, Li et al., 2010). Furthermore, no clinical trials have explored probiotics and prebiotics alone and in combination to assess their potential synergistic benefits for metabolic health. Therefore, we conducted a double-blind, randomized, placebo-controlled, multi-center clinical trial adhering to the GCP principles to investigate the effects of a probiotic (B420) and a prebiotic (LU) on weight management and an extensive panel of mechanistic parameters, including markers of low-grade inflammation, adipose tissue metabolism, bacterial translocation and fecal short-chain fatty acids.

## 2 Materials and Methods

This double-blind, randomized, parallel, placebo-controlled clinical trial was conducted in four clinical research centers in southern Finland: VL-Medi Oy (Helsinki), Clinical Research Services Turku (Turku), FinnMedi Oy (Tampere) and Kerava Health Care Center (Kerava). All research procedures performed in this trial were in strict accordance with a pre-defined protocol, and adhered to international GCP guidelines and the Declaration of Helsinki. The study was approved by the Coordinating Ethics Committee of the Hospital District of Helsinki and Uusimaa and all participants signed informed consent prior to participation. This study is registered in ClinicalTrials.gov with the identifier NCT01978691.

### 2.1 Participants

Eligible participants were 18–65 years old with a body mass index (BMI) between 28.0–34.9 and a waist to hip ratio of ≥ 0.88 for males and ≥ 0.83 for females.

Exclusion criteria were, briefly: diagnosed type 1 or type 2 diabetes or cardiovascular disease, or use of related medication; use of laxatives, fiber supplements or probiotics in the previous 6 weeks; inflammatory disorders and use of immunomodulatory drugs; history of bariatric surgery; use of *anti*-obesity drugs in the previous 3 months; recent (last 2 months) or on-going antibiotic use; excessive use of vitamin D supplementation; active or recent (last 3 months) participation in a weight loss program or weight change of 3 kg during the past 3 months; pregnant or planning pregnancy within 6 months or breastfeeding women; drug or alcohol abuse; and other reasons identified by the Investigator.

### 2.2 Intervention, Compliance and Stability

All study products were obtained from DuPont Nutrition and Health (Madison, WI, USA) in sachets. The study arms were: 1) Placebo, microcrystalline cellulose 12 g/day; 2) B420, 10^10^ CFU/day in 12 g of microcrystalline cellulose (B420); 3) LU, 12 g/day; and 4) B420, 10^10^ CFU/day in 12 g of LU (LU + B420). Microcrystalline cellulose was chosen as placebo because it contains virtually no energy and is less fermented by gut microbes than alternative dietary fibers. Participants were provided with commercially available fruit smoothie products and instructed to mix the contents of one sachet into a 250 ml bottle of smoothie (130 kcal) each day while otherwise maintaining their regular diet. The smoothies were provided to the subjects to mask subtle differences in the study products and to standardize the matrix in which the products were consumed.

Following separate visits for screening and baseline assessments, participants used the study product for six months, during which they came for clinic visits at 2 months, 4 months and 6 months, with in-between telephone contacts to track compliance and potential adverse events. After completing the intervention, participants came for one more follow-up clinic visit one month after the end of the dietary intervention (month 7).

Compliance was monitored with three different methods: 1) Participants were asked to report product intake on a specific check-list, 2) Participants were asked to return all used and unused sachets to the site to count the number of opened sachets per the number of treatment days, and 3) Fecal samples were analyzed for the presence of B420 with qPCR from all participant who returned a fecal sample at the six-month visit. Details of the qPCR detection procedures are reported in the Supplemental Materials and Methods. In the Intention-to-Treat (ITT) population, the average calculated compliance (method 2) was 86% across groups, while in the B420 and LU + B420 groups the bacterial strain was detected in the feces of 82% of participants. The primers used for qPCR were also able to detect certain other *Bifidobacterium animalis* ssp. *lactis* strains such as HN019, Bl-04 and Bi-07, but not Bb12. Four participants (8%) in the Placebo group and seven (15%) in the LU group tested positive in the qPCR assay (Table S1).

Product stability was monitored throughout the study. The minimum target activity for B420 was planned at 1 × 10^10^ CFU/day. The dose of B420 at the time of packaging was 1.4 × 10^10^ CFU/day in the B420 group and 1.3 × 10^10^ CFU/day in the LU + B420 group to account for loss of stability during the study. Sachets returned from the study participants were re-tested for probiotic cell count at the end of the study with the following results: B420 1.1 × 10^10^ CFU/day, and LU + B420 1.1 × 10^10^ CFU/day, demonstrating excellent stability of the study probiotic. There were no contaminations in the Placebo and LU products.

### 2.3 Body Composition-Related Outcomes

The pre-defined primary outcome of the present study was the relative change in body fat mass from baseline to end-of-treatment (6 months). Body fat mass was measured with a dual-energy X-ray absorptiometry (DEXA) scan at qualified private medical centers. In addition, body fat mass and lean body mass were recorded as total and from individual regions of the body (android, gynoid, trunk, legs, arms). Other obesity-related outcomes included body weight and waist and hip circumference, which were measured with calibrated weighing scales and tape measures, respectively.

### 2.4 Clinical Laboratory Outcomes

At the clinic visits (baseline, 2 months, 4 months, 6 months and follow-up), blood was drawn in the morning from fasting participants and sent from all sites to a certified central laboratory (United Medix Laboratories, Finland) for analysis of serum high-sensitivity C-reactive protein (hsCRP), serum glucose, serum insulin, blood glycosylated hemoglobin (HbA1c), serum lipids (total cholesterol, LDL, HDL and triglycerides) and serum cortisol. The homeostatic model assessment of insulin resistance (HOMA-IR) was calculated from fasting glucose and insulin levels ([glucose in mmol/l] * [insulin in mU/l] / 22.5). Serum liver markers (ASAT, ALAT, gamma-glutamyltransferase) were also analyzed at the central laboratory to monitor product safety.

### 2.5 Exploratory Laboratory Outcomes

The following blood biomarkers were analyzed with ELISA: interleukin-6 (IL-6), tumor necrosis factor alpha (TNF-α), interleukin-1beta (IL-1β), plasminogen activator inhibitor 1 (PAI-1), vascular cell adhesion protein 1 (VCAM-1), intracellular adhesion molecule 1 (ICAM-1), *E*-selectin, sCD14, leptin, monocyte chemoattractant protein 1 (MCP-1), adiponectin, zonulin, angiopoietin-like protein 4 (angptl4), oxidized LDL cholesterol (oxLDL) and ApoB-48. The employed assay kits and their performance characteristics (coefficients of variation) are detailed in Supplementary Materials and Methods.

For lipopolysaccharide (LPS) analyses, serum samples were diluted 1:100, heat-treated for 45 min at 60 °C, and assayed in triplicate using the Limulus Amebocyte Lysate (LAL) kinetic chromogenic method (Charles River). The quantitation limit was 48 EU/l.

### 2.6 Analysis of Acidic Fermentation Products in Fecal Samples

Fecal short-chain fatty acids (SCFA) were analyzed with gas chromatography, as detailed in the Supplemental Materials and Methods.

### 2.7 Food Intake and Exercise Measurements

Food intake was measured with a 5-day food diary at baseline, 2 months and 6 months (end of intervention) as detailed in the Supplemental Materials and Methods.

Habitual exercise was captured with a simple three-point questionnaire and calculated into metabolic equivalents (MET h/day), as previously described (Kujala et al., 1998).

### 2.8 Adverse Events

Investigators asked study participants about adverse events at every study visit. All possible adverse events were recorded and coded according to MedDRA. The adverse events were classified by the Investigator according to severity (mild, moderate, severe) and possible relationship to treatment (unrelated, unlikely, possible, probable, not assessable).

### 2.9 Sample Size Determination

A one-way analysis of covariance (ANCOVA) was used to analyze the primary outcome, with baseline body fat mass as covariate. The mean relative change from baseline to 6 months in body fat mass was assumed to be 1% in the active treatment groups compared to the placebo group, and the common standard deviation was expected to be 2%. With these assumptions, 43 participants per group (172 in total) was calculated to give above 80% power to reject a null hypothesis of no difference between the active treatments compared to placebo with a one-way ANOVA, using a 5% level of significance. The power for the ANCOVA model was expected to be similar or slightly higher than for an ANOVA model. In order to compensate for an expected drop-out rate of approximately 23.5%, a total of 225 participants were randomized into the study.

### 2.10 Randomization and Blinding

The randomization scheme (1:1:1:1 allocation) was generated using a computerized procedure into blocks of four randomization codes each. Boxes of investigational product were labeled with the corresponding randomization code and study centers were advised to always use the smallest available randomization code and corresponding study product. The randomization code was generated by the contract research organization Smerud Medical Research Finland Ab Oy (Kirkkonummi, Finland). The participants, the site personnel, the study monitor, the statistician and sponsor's representatives were all blinded to the randomization until the end of the intervention phase, when all data for primary and secondary outcomes, adverse events and compliance had been collected and validated. The study populations were defined prior to breaking the treatment code. Exploratory outcomes were assayed in a blinded fashion, but were not included in the blind data review process.

### 2.11 Statistical Methods

The primary and pre-determined statistical analysis was an ANCOVA to compare changes from baseline to end-of-intervention. Baseline values were used as covariates when they were significant in the model. For the ITT population, the last observation was carried forward for missing observations. All group-wise comparisons were performed using Dunnett's adjustment for multiple testing. A factorial analysis (two-way ANCOVA) was conducted post-hoc after unblinding, because of the lower number of participants in the B420 group compared to the other groups. There was no apparent reason for the higher number of exclusions in the B420 group, since the reasons for exclusion were similar across all groups.

Data for LPS, hsCRP and IL-6 were considered non-normally distributed. Data for hsCRP and IL-6 were analyzed with ANCOVA and Dunnett's adjustment after log-transformation. Data for LPS were analyzed with the Kruskal-Wallis test, with Dwass, Steel, Critchlow-Fligner analysis of between-group comparisons. Correlations between changes from baseline to end-of-study for the different markers were calculated with Spearman correlation.

All data were analyzed with SAS version 9.3 using a significance level of 0.05.

## 3 Results

### 3.1 Recruitment and Participant Flow

All 225 participants were randomized at four centers in southern Finland between December 2013 and October 2014, and the last participant completed the last follow-up visit in May 2015. Study participants were randomized to four groups for a six-month intervention and a one-month follow-up period: 1) Placebo, 12 g/day of microcrystalline cellulose; 2) LU, 12 g/day; 3) B420, 10^10^ Colony Forming Units (CFU) /day in 12 g microcrystalline cellulose; 4) LU + B420, 10^10^ CFU/day of B420 in 12 g/day LU.

**Fig. 1:**
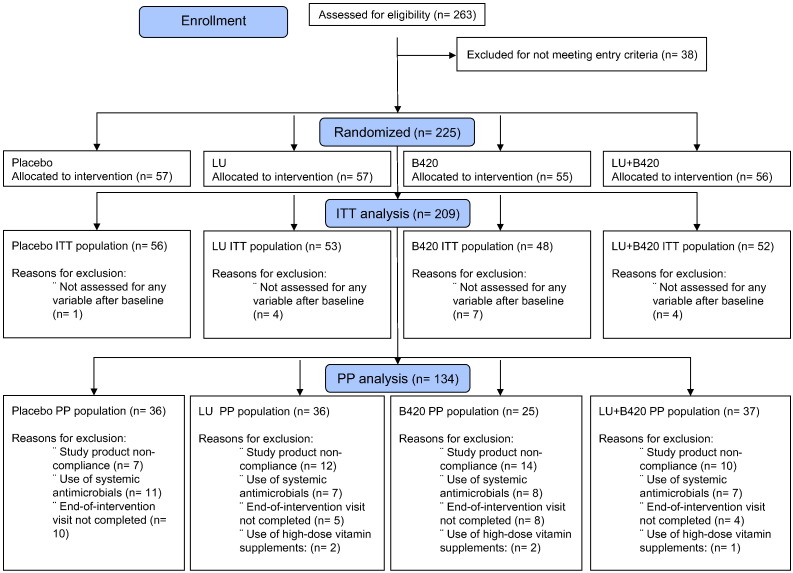
Participant flow. Before unblinding the study, participants were divided into an Intention-to-Treat (ITT) population and a Per Protocol (PP) population according to adherence to the study protocol.

The study populations and reasons for exclusions are shown in Fig. 1. Of the 225 randomized participants, the Investigator excluded one person from the study due to very high cholesterol levels, before the participant had taken any study product. Therefore, the total population analyzed for adverse events included 224 participants. The ITT population contained all 209 participants who were analyzed for any variable after the baseline visit, whereas the Per Protocol (PP) population included, as defined in the protocol, all 134 participants who completed the study without major protocol violations and who had at least more than one study visit completed. A Blind Data Review process prior to unblinding the study detailed the major protocol violations as follows: 1) < 80% of study product compliance or > 7 consecutive days of non-compliance; 2) Baseline or 6-month visit not completed; 3) Participant randomized against inclusion and exclusion criteria (e.g. use of high-dose vitamin D supplements); and 4) Use of systemic antibiotics during the intervention period. In addition, three participants were excluded from the PP population for body composition measurements for not completing the measurement within ten days after discontinuing treatment. None of the participants were reallocated to another group for analysis.

The demographics of the ITT and PP populations are shown in Table 1 and were similar among the different treatment groups. Statistical conclusions for the ITT and PP populations differed greatly, and therefore drop-outs and protocol violations—which are common especially in long intervention studies such as this—were considered to have a significant impact on the outcomes. Consequently, the present report is mainly focused on the protocol-compliant PP population. Main outcomes for the ITT population can be found in Table S2.

### 3.2 Adverse Events and Safety Parameters

All symptoms occurring during the study were documented and rated for severity and potential relationship with the study products. There were no major differences in the distribution of adverse events across groups. Over the course of 7 months, 199 of 224 study participants reported at least one adverse event, out of which 133 were potentially product-related. Adverse events are summarized in Table S3. Gastrointestinal symptoms were generally mild, and the median duration of the cases of diarrhea and loose stools was three days. There were no changes in measured safety parameters: blood pressure, heart rate and liver enzymes (Table S3).

### 3.3 B420 with or without LU Controls Body and Trunk Fat Mass, and Show Synergistic Effects on Lean Body Mass

**Fig. 2:**
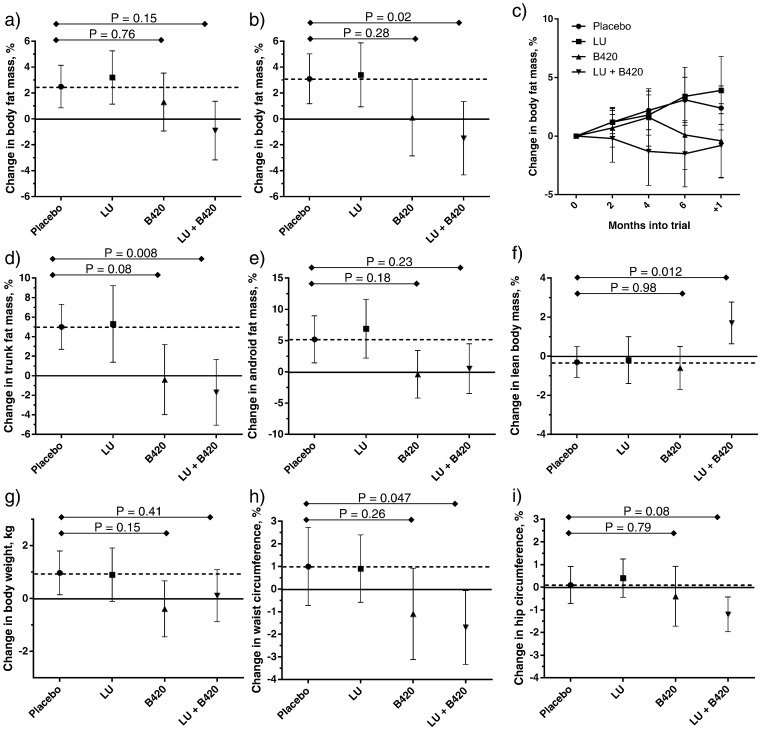
Weight management outcomes. Change in total body fat mass from baseline to end of study (6 months of intervention) in the Intention-to-Treat (a) and Per Protocol (PP) (b) populations. Evolution of body fat mass in the PP population during intervention and at follow-up (c). Changes in trunk fat mass (d), android fat mass (e), lean body mass (f), body weight (g), waist circumference (h) and hip circumference (i) from baseline to end of study in the PP population. Body composition in panels a-f was measured with dual-energy X-ray absorptiometry. Results are expressed as mean ± 95% CI. Results were analyzed with ANCOVA and Dunnett's pairwise comparisons corrected for multiple testing. In the ITT population, the last observation was carried forward for the statistical analysis of participants who withdrew from the study. Solid line shows baseline; dotted line shows the level of the Placebo group. N of participants included in the statistical analysis in the Intention-to-Treat population: Placebo *n* = 53, LU *n* = 51, B420 *n* = 47, LU + B420 *n* = 48. N for the PP population: Placebo *n* = 35–36, LU *n* = 35–36, B420 *n* = 24–25, LU + B420 *n* = 37. Overall ANCOVA as follows: a) *P* = 0.46, b) *P* = 0.095, d) *P* = 0.036, e) *P* = 0.23, f) *P* = 0.30, g) *P* = 0.13, h) *P* = 0.10, i) *P* = 0.31.

The primary outcome of this study was the relative change in body fat mass from baseline to the end of the intervention period (6 months). Body composition was measured with DEXA every two months during the intervention and at the follow-up after one month. In the ITT population, there were no statistically significant differences between the active treatment groups and Placebo in the changes in total body fat mass (Fig. 2A). In the PP population, however, the LU + B420 group showed a significant reduction in the change in total body fat mass compared to Placebo (Fig. 2B), resulting in an average difference of 1.4 kg in total body fat between the groups. The effect seemed to persist throughout the one-month wash-out period (Fig. 2C). The difference in body fat mass was most noticeable in the trunk area and demonstrated a similar, although non-significant pattern in the abdominal (i.e. android) area (Fig. 2D–E). In other regions of the body LU + B420 had no effect. LU and B420 (alone) were not statistically different from Placebo in any of the above mentioned variables. For absolute values in all measured areas of the body in the PP population, please refer to Table S4. Changes in total body fat mass per the following subgroups are shown in Fig. S1: men vs. women, low vs. high baseline fat intake, and those in the ITT population who used systemic antimicrobials during the study. Markers of glucose metabolism are presented in Table S5, and cardiovascular biomarkers in Table S6.

Because of the lower number of observations in the B420 group (*n* = 24) compared to the other groups (*n* = 35–37), we decided to conduct a post-hoc factorial analysis to evaluate the independent effects of B420 and LU in the PP population. In the PP population, there were significant differences between the B420 and Placebo groups in their changes in total body fat mass (*P* = 0.002), trunk fat mass (*P* = 0.0002) and android fat mass (*P* = 0.004), indicating that the probiotic itself could be effective for controlling fat mass, especially in the abdominal region. LU had no independent effects on these parameters.

In addition, the results for lean body mass suggested synergy between the two investigational products, LU and B420. Although neither of the products alone had an effect on lean body mass, their combination significantly increased lean body mass compared to Placebo (Fig. 2F), and the difference developed as a gradual increase from baseline, although there were no changes in the physical exercise habits in any of the groups (Fig. S2).

### 3.4 B420 With or Without LU Reduces Waist Circumference Compared to Placebo

The differences in body fat mass outcomes were not completely reflected in body weight (Fig. 2G). In fact, only the B420 group showed any trend towards a reduction in body weight (*P* = 0.15) in the PP population, whereas in the LU + B420 group, the reduction in body fat mass was counterbalanced with an increase in lean body mass. In the factorial analysis, there was a significant effect for B420 (*P* = 0.03), but no effect for LU (*P* = 0.80) compared to placebo in the PP population. The LU + B420 group also showed a 2.7% (2.6 cm) reduction in waist circumference (*P* = 0.047) (Fig. 2H) and a trend (*P* = 0.079) towards a 1.3% (1.4 cm) smaller hip circumference compared to Placebo (Fig. 2I). According to the factorial analysis, there was a significant 2.4% difference in the change in waist circumference between B420 and Placebo (− 2.4 cm compared to Placebo) (*P* = 0.004).

### 3.5 Six-Month Supplementation with B420 and LU + B420 Reduces Energy Intake Compared to Placebo

We measured dietary intake with 5-day food diaries at baseline and at 2 and 6 months after study start. There were no differences between the groups in baseline dietary intake (Table S7). B420 and LU + B420 reduced energy intake by approximately 300 kcal/day and 210 kcal/day compared to Placebo (Table 2). This was associated with a reduced intake of dietary fat and fiber in the B420 group, but without differences in the relative proportion of fat in the diet.

B420 also increased plasma cortisol concentrations (Fig. S3), which has been suggested to increase energy expenditure and fat oxidation (Brillon et al., 1995) and to suppress inflammation. Increased plasma cortisol may also be related to an adverse stress response (Zunszain et al., 2011), however, there was only one psychiatric-related adverse event in the B420 group: a mild worsening of depression, which also occurred in one participant in the Placebo group. The investigator evaluated both cases as unrelated to the study product.

### 3.6 Changes in Fat Mass Correlate with Circulating Zonulin and hsCRP

**Fig. 3:**
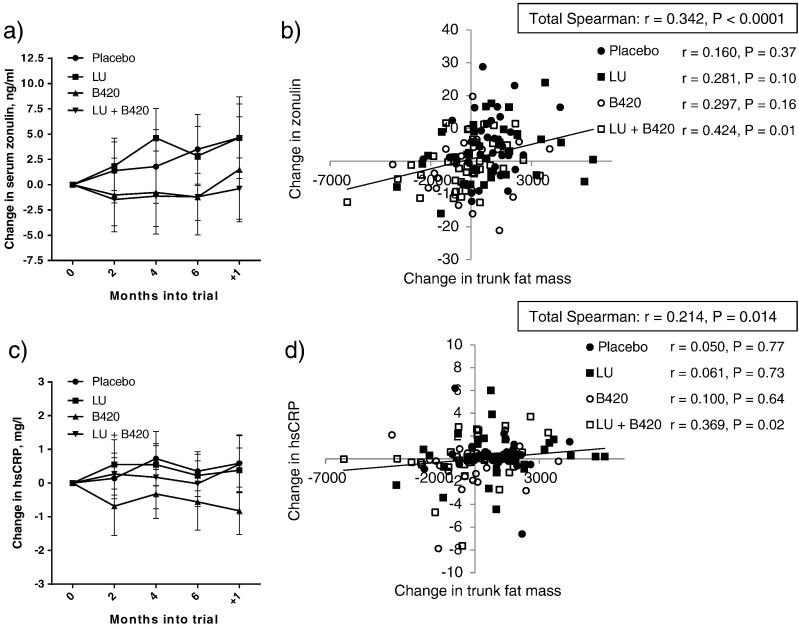
Correlation between serum zonulin, serum hsCRP and trunk fat mass. Evolution of zonulin (a) and hsCRP (c), and correlations between zonulin (b) and hsCRP (d) with trunk fat mass as changes from baseline to end-of-intervention (6 months) in the Per Protocol population. Panels a) and c) display changes from baseline as mean ± 95% confidence intervals at each time point. Placebo *n* = 35–36, LU *n* = 35–36, B420 *n* = 24–25, LU + B420 *n* = 36–37.

Levels of circulating zonulin, a potential marker of intestinal permeability (Fasano et al., 2000, Moreno-Navarrete et al., 2012), seemed to remain consistently lower throughout the study in the B420 and LU + B420 groups compared to Placebo and LU (Fig. 3A), which was reflected as a trend towards a difference between B420 and Placebo in the factorial analysis (*P* = 0.063) (Table 3). Changes in zonulin were also statistically significantly correlated with changes in trunk fat mass (*r* = 0.349, *P* < 0.0001) (Fig. 3B), indicating a potential mechanistic link between the two. This correlation was only evident in the LU + B420 group.

Changes in hsCRP were somewhat similar to the changes in zonulin (Fig. 3C). Although there were no differences between groups in the primary analysis, a factorial analysis showed a tendency towards a reduction in hsCRP in B420 vs. Placebo (*P* = 0.073) (Table 3). Changes in hsCRP were also significantly correlated with changes in trunk fat mass (*r* = 0.217, *P* = 0.012) (Fig. 3D), but again only in the LU + B420 group. Changes in hsCRP were also significantly correlated with changes in zonulin (Spearman correlation *r* = 0.199, *P* = 0.021).

LU + B420 appeared to increase LPS level in plasma compared to Placebo (Kruskal-Wallis *P* = 0.009), although the changes were small and LPS level remained below or close to the detection limit in most participants. There was no effect on soluble cluster of differentiation 14 (sCD14), a co-receptor of LPS (Table 3), underlining the lack of inflammatory response to the slightly increased LPS levels. There were also no differences in IL-6 levels (Table 3). Concentrations of TNF-α and IL-1β were below the lower limit of quantitation in 95 and 97% of the samples, respectively.

The corresponding outcomes for the ITT population are shown in Table S8. Adipose tissue-related biomarkers are shown in Table S9.

### 3.7 Increased Production of Bacterial Metabolites in Feces

In the factorial analysis, B420 increased the concentration of fecal propionic acid, butyric acid and valeric acid (*P* < 0.05) (Table 4, data for ITT population shown in Table S10.), which indicated an increased metabolism of non-digestible polysaccharides and suggests changes in the composition of the gut microbiota. A tendency for similar changes was noted for acetic acid, although the factorial analysis was not statistically significant (*P* = 0.13). In the primary statistical analysis, only valeric acid was significantly increased by B420; there were no other statistically significant differences between the groups. Total fecal SCFA concentrations were also increased by B420 in the factorial analysis.

## 4 Discussion

Over the past decade, advances in science have established a link between gut microbes and obesity, raising interest in developing probiotics and prebiotics for weight management. This study shows consistent results towards an improvement in weight management -related parameters in the probiotic and synbiotic groups, including total body fat mass, trunk fat mass, waist circumference, and energy intake in the Per Protocol population. Furthermore, our study demonstrates synergy between a probiotic and a prebiotic fiber supplement in promoting lean body mass accumulation. We also present results on biomarkers associated with gut barrier function, reduced inflammatory tone and intestinal microbiota metabolism that suggest a potential mechanism of action for B420 and LU + B420 in reducing body fat mass.

We have previously shown that B420 reduces body fat mass gain in mice that are fed a high-fat diet (Stenman et al., 2014). In the current study, only the LU + B420 group significantly reduced total body fat mass compared to Placebo in the PP population, while there were no differences in the ITT population. A post-hoc factorial analysis showed that B420 alone had a significant effect in both the ITT and PP population. The differences between the treatment groups were consistent across several outcomes, providing further support to the previous results obtained in experimental animals.

We conducted statistical analyses on both, ITT and PP populations, with different conclusions. In the ITT populations we used for missing observations the Last Observation Carried Forward method that is a widely accepted approach, but can alter the conclusions of very long studies, which are prone to drop-outs and protocol violations. The findings of the PP population can be considered as being better representative of the effect of the investigated products, but they, in turn, are prone to bias from participant drop-outs. In this study, our PP population included 64% of the ITT population, mainly due to drop-outs, non-compliance and use of systemic antimicrobials. This weakens the statistical power in the PP population and warrants further studies to support the present findings.

In the current study, the study participants in the Placebo group gained approximately 1 kg (1.1%) of weight compared to baseline. This weight gain was not due to increased energy intake from the smoothie vehicle, as energy intake did not change in the Placebo group (− 23 kcal). On the contrary, the LU + B420 and B420 groups showed considerable reductions in their energy intake (− 230 and − 320 kcal/day, respectively), while body weight and body fat mass did not differ from baseline, suggesting that B420 and LU + B420 normalized energy balance by reducing energy intake to a level that maintains, rather than increases, body fat mass.

According to the Helsinki Health Study, steady weight gain over time is rather common among an average Finnish middle-aged population (Loman et al., 2013), as approximately 30% gained at least 5 kg weight during a 5–7-year follow-up period. Our overweight and obese study population (BMI 28–34.5) may have been more prone to weight gain than the average population in the Helsinki Health Study.

Four clinical studies involving healthy, overweight and/or obese participants have investigated the effects of micro-organisms on weight management or body fat mass (Kadooka et al., 2010, Kadooka et al., 2013, Minami et al., 2015, Sanchez et al., 2014). The effect size seen in the current study (6.7% difference in trunk fat between LU + B420 and Placebo) is quite comparable to that seen in 12-week interventions with *Lactobacillus gasseri* SBT2055 where abdominal fat was reduced by approximately 4.5% compared to placebo (Kadooka et al., 2010, Kadooka et al., 2013). It is difficult to compare the employed statistical methodology, however, because it remains unclear which participants were included in the analyses. In comparison, intake of *Bifidobacterium breve* B-3 reduced body fat mass by 0.6 kg compared to placebo within 12 weeks (Minami et al., 2015). It should also be noted that the study participants in these three studies were Japanese whereas the present study was conducted on a Western population.

In a Canadian study population, a combination of *Lactobacillus rhamnosus* CGMCC1.3724 (LPR), inulin and oligofructose helped to further reduce weight (2.7 kg) and total body fat mass (1.8%) during a weight-loss and weight maintenance period, but only in women (Sanchez et al., 2014). In the present study, we did not find marked differences between the observed effects in men and women.

In terms of effect size, it is difficult to compare probiotics to pharmaceuticals, because the latter have been tested for weight loss instead of fat mass loss, and they are accompanied by a weight-loss program. However, the reported changes in body weight at six months compared to placebo have been: − 3.5 kg for orlistat (Torgerson et al., 2004), − 5.5% for liraglutide (Pi-Sunyer et al., 2015), and − 3.2% for locaserin (O'neil et al., 2012), compared to − 1.4 kg (− 4.5%) with LU + B420 and − 1.0 kg (− 3.0%) with B420 in fat mass in the present study. It should be noted that probiotics are taken orally once per day, while orlistat is taken multiple times per day and liraglutide requires a daily subcutaneous injection. Therefore, probiotics and synbiotics could be a safe and convenient, non-pharmaceutical support for controlling body fat mass, especially prophylactically.

The results of this study showed that changes in circulating zonulin levels correlated with changes in trunk fat mass, but only in the LU + B420 group, indicating a potential mechanistic relationship between zonulin and trunk fat mass. Also, B420 tended to reduce levels of circulating zonulin. Zonulin is the human analogue of the zonula occludens toxin produced by *Vibrio cholera* (Wang et al., 2000, Fasano et al., 2000). It selectively increases intestinal permeability in jejunum and ileum (Wang et al., 2000), and its release is induced by certain gut microbes, especially pathogens (El Asmar et al., 2002), indicating that zonulin could act as a mechanistic link between changes in the gut microbiota and gut barrier function.

When measured from circulation, zonulin has been linked with insulin resistance (Moreno-Navarrete et al., 2012), type 2 diabetes (Zhang et al., 2014), higher BMI (Moreno-Navarrete et al., 2012) and non-alcoholic fatty liver disease (Pacifico et al., 2014). The link between circulating zonulin and intestinal zonulin remains somewhat elusive, since circulating zonulin might not originate only from the gut (Wang et al., 2000). Nevertheless, decreased circulating zonulin is associated with a decreased lactulose/mannitol ratio, an indicator of intestinal permeability (Russo et al., 2012, Liu et al., 2013), as well as fewer postoperative infection complications (Liu et al., 2013). Furthermore, type 1 diabetic patients and their relatives were found to have increased circulating zonulin levels that correlated with intestinal permeability (Sapone et al., 2006). According to these findings, it is plausible that the changes in circulating zonulin, as seen in the present study, are related to an improvement in gut barrier function.

Improved gut barrier function is hypothesized to reduce metabolic endotoxemia, especially the concentration of highly inflammatory LPS, and reduce low-grade inflammation (Burcelin et al., 2009). Our earlier studies have shown that B420 improves epithelial barrier function in cell culture (Putaala et al., 2008) and reduces epithelial translocation of *E. coli* as well as circulating LPS levels in mice (Amar et al., 2011, Stenman et al., 2014). We have also recently shown that LU + B420 modulates the intestinal immune system in mice and changes the microbial DNA signature in adipose tissue, which were associated with improvements in glucose metabolism (Garidou et al., 2015).

In this present study we saw a subtle increase in plasma LPS levels without activation of inflammatory responses. LPS levels were below or close to the detection limit both at baseline and at the end of intervention, questioning the clinical relevance of this LPS increase. However, this finding could point to a different manifestation of metabolic endotoxemia between mice and humans. The LAL methodology detects total LPS level in a sample and does not specify which bacterial species the LPS molecules originate from. It has been reported that the LPS levels detected with the LAL method may considerably differ from their cytokine-producing potency (Gutsmann et al., 2010). Thus, although the level of total LPS was increased with LU + B420 treatment, its quality and inflammatory potency may have also been changed by the treatment. This hypothesis was supported by the tendency towards reduced hsCRP levels.

We did not see treatment-associated reductions in blood glucose, HbA1c, insulin or HOMA-IR in the present study. Although we recruited overweight and obese participants, baseline laboratory values indicated that the participants had normal fasting glucose (average 5.18–5.24 mmol/l) and insulin (average 7.84–9.07 mU/l) levels, leaving very little room for improvement. Previously, we have shown improvement in glucose metabolism with both B420 alone (Stenman et al., 2014, Stenman et al., 2015b) and in combination with LU (Garidou et al., 2015). The treatments should be tested in populations with impaired glucose metabolism or in patients with type 2 diabetes.

This randomized, double-blind, placebo-controlled clinical intervention trial in overweight and obese adults presents preliminary clinical evidence that the probiotic B420 with or without Litesse Ultra polydextrose can reduce body fat mass, waist circumference, energy intake and body weight compared to placebo. Our findings are of special interest to populations who struggle to control their weight in a Western cultural environment. Additionally, B420 and LU seem to have synergistic effects in increasing lean body mass. LU alone had no effect on the parameters tested. Mechanistically, the reduction of body fat mass might be related to circulating zonulin, a potential marker of gut barrier function, and attenuated low-grade inflammation, which both support previous findings from experimental animals. Further clinical trials are warranted to confirm these effects in larger participant populations and to further elucidate the underlying mechanisms.

## Funding Sources

This study was fully funded by DuPont Nutrition & Health. DuPont, Smerud and the Principal Investigator were jointly responsible for drafting the study protocol and reports as well as interpretation of the data. The lead author of this manuscript was employed at DuPont.

## Conflicts of Interest

This study was fully funded by DuPont Nutrition & Health. Each of the authors or their respective organizations were financially compensated by DuPont for their contribution in the study. In addition, M.S. is a shareholder of Clinical Research Services Turku, and M.C. and R.B. are founders and shareholders of Vaiomer.

## Author Contributions

Conceptualization, R.B., S.L.; Methodology, L.K.S., M.J.L., N.M., H.K.S., A.R., and S.L.; Formal Analysis, N.M.; Investigation, L.K.S., M.J.L., N.M., J.E.C., N.Y., M.T.S., M-L.L., J.L., D.A., and M.S.; Resources, M.C., M-L.L., J.L., D.A., M.S., and S.L.; Writing—Original Draft, L.K.S.; Writing—Review & Editing, M.J.L., N.M., N.Y., M.T.S., R.B., H.K.S., M.S. and S.L.; Visualization, L.K.S. and N.M; Supervision, R.B., H.K.S, A.R., and S.L.; Project Administration, L.K.S.

## Figures and Tables

**Table 1:** Baseline demographics of the Intention-to-Treat and Per Protocol populations.

		Placebo	LU	B420	LU + B420	Total
Intention-to-Treat	*n*	56	53	48	52	209
Gender	n, Male/Female	12/44	12/41	9/39	9/43	42/167
Ethnicity	n, Caucasian/Other	55/1	52/1	48/0	51/1	206/3
Age, years	mean ± SD	49.9 ± 8.5	48.8 ± 10.5	50.6 ± 10.6	47.0 ± 11.1	49.1 ± 10.2
Weight, kg	mean ± SD	88.5 ± 12.2	89.4 ± 9.1	88.7 ± 9.3	87.8 ± 11.0	88.6 ± 10.4
BMI, kg/m^2^	mean ± SD	31.2 ± 2.2	31.2 ± 1.6	31.5 ± 2.2	31.3 ± 2.0	31.3 ± 2.0
Waist circumference, cm	mean ± SD	103.0 ± 8.4	103.4 ± 6.1	102.6 ± 6.9	102.5 ± 6.9	102.9 ± 7.1
Blood glucose, mmol/l	mean ± SD	5.24 ± 0.50	5.20 ± 0.62	5.18 ± 0.43	5.19 ± 0.42	5.20 ± 0.50
Total Cholesterol, mmol/l	mean ± SD	5.23 ± 0.87	5.22 ± 1.00	5.26 ± 1.08	5.50 ± 0.96	5.30 ± 0.97
LDL Cholesterol, mmol/l	mean ± SD	3.10 ± 0.77	3.08 ± 0.82	3.25 ± 0.91	3.38 ± 0.89	3.20 ± 0.85
Triglycerides, mmol/l	mean ± SD	1.22 ± 0.61	1.27 ± 0.53	1.27 ± 0.56	1.29 ± 0.61	1.26 ± 0.58
Per-Protocol	*n*	36	36	25	37	134
Gender	n, Male/Female	10/26	8/28	7/18	6/31	31/103
Ethnicity	n, Caucasian/Other	36/0	35/1	25/0	37/0	133/1
Age, years	mean ± SD	48.3 ± 8.6	48.6 ± 10.9	49.1 ± 11.9	47.1 ± 10.9	48.2 ± 10.4
Weight, kg	mean ± SD	88.7 ± 12.5	89.7 ± 9.4	88.9 ± 10.3	87.7 ± 11.3	88.7 ± 10.9
BMI, kg/m^2^	mean ± SD	31.0 ± 2.2	31.2 ± 1.6	30.9 ± 1.9	31.2 ± 2.0	31.1 ± 1.9
Waist circumference, cm	mean ± SD	102.1 ± 7.5	103.3 ± 6.6	103.3 ± 7.5	102.1 ± 7.3	102.7 ± 7.2
Blood glucose, mmol/l	mean ± SD	5.21 ± 0.54	5.18 ± 0.68	5.16 ± 0.35	5.22 ± 0.42	5.20 ± 0.52
Total Cholesterol, mmol/l	mean ± SD	5.16 ± 0.86	5.24 ± 1.01	5.14 ± 0.86	5.58 ± 0.90	5.29 ± 0.92
LDL Cholesterol, mmol/l	mean ± SD	3.05 ± 0.75	3.12 ± 0.73	3.12 ± 0.66	3.39 ± 0.84	3.17 ± 0.76
Triglycerides, mmol/l	mean ± SD	1.20 ± 0.67	1.25 ± 0.54	1.33 ± 0.58	1.36 ± 0.66	1.28 ± 0.62

**Table 2:** Changes in food intake during the study, as assessed with 5-day food diaries (Per Protocol population).

		Energy intake (kcal/day)				Fat intake (g/day)				Relative fat intake (% kcal)				Fiber intake (g/day)			
		*Absolute*		*Change from baseline*		*Absolute*		*Change from baseline*		*Absolute*		*Change from baseline*		*Absolute*		*Change from baseline*	
*Overall P*				*P = 0.005*				*P = 0.008*				*P = 0.058*				*P = 0.055*	
*Group*	*Visit*	n	Mean ± SD	n	Mean ± SD	n	Mean ± SD	n	Mean ± SD	n	Mean ± SD	n	Mean ± SD	n	Mean ± SD	n	Mean ± SD
Placebo^a^	*Baseline*	33	2240 ± 510	0	–	33	96 ± 32	0	–	33	38 ± 7	0	–	33	21 ± 6.6	0	–
	*Month 2*	34	2270 ± 570	31	57 ± 500	34	90 ± 32	31	− 4.9 ± 32	34	35 ± 7	31	− 2.7 ± 6	34	20 ± 7.3	31	− 1.3 ± 7
	*Month 6*	36	2180 ± 460	33	− 23 ± 600	36	92 ± 31	33	− 2.2 ± 43	36	37 ± 7	33	− 0.6 ± 8	36	20 ± 5.6	33	− 1.2 ± 6
LU	*Baseline*	33	2210 ± 650	0	–	33	92 ± 29	0	–	33	38 ± 5	0	–	33	22 ± 7.9	0	–
	*Month 2*	35	2170 ± 640	33	− 15 ± 430	35	93 ± 31	33	2.2 ± 23	35	39 ± 5	33	0.9 ± 6	35	20 ± 9.2	33	− 1.5 ± 7
	*Month 6*	34	2000 ± 520	33	− 200 ± 510	34	80 ± 27	33	− 12 ± 24	34	35 ± 4	33	− 2.0 ± 6	34	21 ± 9.3	33	− 1.1 ± 6
B420	*Baseline*	24	2200 ± 380	0	–	24	94 ± 22	0	–	24	38 ± 5	0	–	24	24 ± 6.6	0	–
	*Month 2*	22	2130 ± 380	22	− 120 ± 250	22	87 ± 26	22	− 9.7 ± 18	22	36 ± 7	22	− 2.4 ± 6	22	19 ± 5.7	22	− 5.6 ± 5
	*Month 6*	22	1900 ± 370	22	− 320 ± 300⁎	22	74 ± 22	22	− 22 ± 19⁎	22	35 ± 6	22	− 4.0 ± 6	22	19 ± 6.8	22	− 5.6 ± 6⁎
LU + B420	*Baseline*	35	2090 ± 640	0	–	35	87 ± 32	0	–	35	37 ± 6	0	–	35	20 ± 6.2	0	–
	*Month 2*	34	1990 ± 440	33	− 110 ± 530	34	79 ± 26	33	− 8.6 ± 29	34	35 ± 7	33	− 1.7 ± 7	34	16 ± 4.0	33	− 4.2 ± 6
	*Month 6*	31	1870 ± 440	29	− 230 ± 640⁎	31	74 ± 31	29	− 10 ± 29	31	35 ± 6	29	− 1.2 ± 5	31	16 ± 5.1	29	− 3.9 ± 5

⁎ = Significant difference from Placebo, *P* < 0.05 (Dunnett's test, corrected for multiple comparisons). Only changes from baseline to month 6 were statistically compared between groups.

a Women with energy intake < 80% and men with energy intake < 85% of basal metabolic rate excluded from dietary intake analyses. The energy content of the smoothie vehicle is included in the results.

**Table 3:** Changes in markers of endotoxemia and low-grade inflammation (Per Protocol population).

		Placebo	LU	B420	LU + B420	*Overall P*	*Factorial P B420*	*Factorial P LU*
*Outcome*		*Mean* *±* *SD*	*Mean* *±* *SD*	*Mean* *±* *SD*	*Mean* *±* *SD*			
	*n*	35–36	35–36	25	37			
Zonulin (ng/ml)	*Baseline*	56.5 ± 12.6	55.5 ± 9.1	58.4 ± 11.4	64.6 ± 14.2			
	*Month 6*	59.7 ± 10.9	58.4 ± 12.0	57.1 ± 8.3	63.4 ± 13.0			
	*Δ (ng/ml)*	+ 3.5 ± 10.0	+ 2.8 ± 8.6	− 1.2 ± 9.1	− 1.2 ± 7.0	0.10	0.063	0.84
ApoB-48 (μg/ml)	*Baseline*	11.0 ± 7.2	9.5 ± 6.9	11.1 ± 5.5	9.8 ± 4.9			
	*Month 6*	11.0 ± 6.1	9.1 ± 5.4	12.6 ± 7.5	10.4 ± 5.7			
	*Δ (*μg*/ml)*	+ 0.07 ± 5.7	− 0.44 ± 5.3	+ 1.53 ± 6.5	+ 0.56 ± 4.0	0.74^a^	0.26^a^	0.092^a^
hsCRP (mg/l)	*Baseline*	1.73 ± 1.4	2.20 ± 2.3	2.78 ± 2.6	2.58 ± 2.6			
	*Month 6*	2.08 ± 1.3	2.43 ± 2.30	2.22 ± 2.6	2.56 ± 2.6			
	*Δ (mg/l)*	+ 0.35 ± 1.7	+ 0.23 ± 1.8	− 0.56 ± 2.0	− 0.02 ± 2.0	0.13^a^	0.073^a^	0.84^a^
LPS (EU/l)	*Baseline*	65 ± 115	120 ± 206	43 ± 61	39 ± 43			
	*Month 6*	39 ± 26	101 ± 365	51 ± 76	48 ± 42			
	*Δ (EU/l)*	− 26 ± 108	− 13 ± 341	+ 7.1 ± 39	+ 9.1 ± 40⁎	0.007^b^	0.094^a^	0.22^a^
sCD14 (μg/ml)	*Baseline*	1.62 ± 0.39	1.82 ± 0.43	1.80 ± 0.37	1.77 ± 0.49			
	*Month 6*	1.80 ± 0.61	1.87 ± 0.49	1.79 ± 0.54	1.75 ± 0.43			
	*Δ (*μg*/ml)*	+ 0.18 ± 0.6	+ 0.06 ± 0.5	− 0.01 ± 0.5	− 0.03 ± 0.5	0.43	0.24	0.78
IL-6 (pg/ml)	*Baseline*	10.5 ± 19	12.8 ± 20	9.0 ± 10	17.5 ± 54			
	*Month 6*	9.2 ± 16	12.3 ± 13	11.2 ± 13	8.0 ± 10			
	*Δ (pg/ml)*	− 1.2 ± 10	− 0.54 ± 15	+ 2.2 ± 13	− 9.5 ± 47	0.72^a^	0.42^a^	0.64^a^

⁎ Significant difference from Placebo, *P* < 0.05 (Dunnett's test, corrected for multiple comparisons). Only relative changes from baseline to month 6 were statistically compared between groups.

a Analyses on log-transformed data.

b Non-parametric analyses.

**Table 4:** Changes in bacterial metabolites in feces in the Per Protocol population.

		Placebo	LU	B420	LU + B420	*Overall P*	*Factorial P B420*	*Factorial P LU*
		Mean ± SD (μmol/g)	*Mean* *±* *SD (*μmol/g)	*Mean* *±* *SD (*μmol/g)	*Mean* *±* *SD (*μmol/g)			
	*n*	36	35–36	24	35–37			
Acetic acid	*Baseline*	42.3 ± 16	43.0 ± 18	38.5 ± 21	43.7 ± 18			
	*Month 6*	37.1 ± 14	37.6 ± 14	39.8 ± 14	41.3 ± 17			
	*Δ*	− 5.20 ± 14	− 5.76 ± 18	+ 1.34 ± 24	− 2.60 ± 18	0.34	0.13	0.99
Propionic acid	*Baseline*	11.8 ± 5.1	12.6 ± 5.7	10.7 ± 5.2	12.6 ± 5.9			
	*Month 6*	9.9 ± 4.0	10.6 ± 5.6	11.6 ± 4.6	12.3 ± 5.9			
	*Δ*	− 1.91 ± 4.1	− 2.00 ± 6.0	+ 0.92 ± 5.8	− 0.31 ± 7.0	0.095	0.025	0.79
Butyric acid	*Baseline*	11.5 ± 6.5	10.8 ± 6.2	11.7 ± 8.6	11.7 ± 6.3			
	*Month 6*	10.2 ± 6.0	9.2 ± 4.8	13.1 ± 9.4	10.3 ± 5.4			
	*Δ*	− 1.33 ± 6.2	− 1.58 ± 5.9	+ 1.35 ± 13	− 1.21 ± 7.2	0.45	0.0497	0.095
Valeric acid	*Baseline*	1.6 ± 0.95	1.5 ± 1.1	1.6 ± 1.1	1.9 ± 0.9			
	*Month 6*	1.3 ± 0.81	1.4 ± 1.0	1.8 ± 1.3	1.5 ± 0.9			
	*Δ*	− 0.31 ± 0.9	− 0.08 ± 1.4	+ 0.26 ± 1.4⁎	− 0.34 ± 1.0	0.083	0.046	0.43
Lactic acid	*Baseline*	0.81 ± 0.89	0.67 ± 0.45	0.71 ± 1.04	0.65 ± 0.82			
	*Month 6*	0.65 ± 0.69	0.88 ± 0.86	1.40 ± 2.69	0.60 ± 0.62			
	*Δ*	− 0.16 ± 0.8	+ 0.22 ± 0.8	+ 0.68 ± 2.2	+ 0.02 ± 0.7	0.14^a^	0.85^a^	0.58^a^
Branched-chain fatty acids^b^	*Baseline*	3.86 ± 2.2	3.37 ± 2.2	3.70 ± 2.2	4.21 ± 2.0			
	*Month 6*	2.77 ± 1.1	3.02 ± 1.5	3.76 ± 3.6	3.08 ± 1.8			
	*Δ*	− 1.09 ± 2.2	− 0.32 ± 2.7	+ 0.06 ± 3.6	− 1.20 ± 1.9	0.11	0.13	0.52
Total	*Baseline*	71.9 ± 25	71.9 ± 28	66.9 ± 33	74.7 ± 28			
	*Month 6*	61.9 ± 22	62.7 ± 23	71.5 ± 27	69.1 ± 26			
	*Δ*	− 10.0 ± 23	− 9.5 ± 28	+ 4.6 ± 41	− 5.6 ± 31	0.19	0.049	0.68

⁎ = Significant difference from Placebo, *P* < 0.05 (Dunnett's test, corrected for multiple comparisons). Only relative changes from baseline to month 6 were statistically compared between groups.

a Non-parametric analyses.

b Sum of isobutyric acid, isovaleric acid and 2-methyl-butyric acid.

## Appendix A Supplementary data

Supplementary material Image 1

## References

[bb0005] Amar J., Chabo C., Waget A., Klopp P., Vachoux C., Bermudez-Humaran L.G., Smirnova N., Berge M., Sulpice T., Lahtinen S., Ouwehand A., Langella P., Rautonen N., Sansonetti P.J., Burcelin R. (2011). Intestinal mucosal adherence and translocation of commensal bacteria at the early onset of type 2 diabetes: molecular mechanisms and probiotic treatment. EMBO Mol. Med..

[bb0010] Brillon D.J., Zheng B., Campbell R.G., Matthews D.E. (1995). Effect of cortisol on energy expenditure and amino acid metabolism in humans. Am. J. Phys..

[bb0015] Brun P., Castagliuolo I., Di Leo V., Buda A., Pinzani M., Palu G., Martines D. (2007). Increased intestinal permeability in obese mice: new evidence in the pathogenesis of nonalcoholic steatohepatitis. Am. J. Physiol. Gastrointest. Liver Physiol..

[bb0020] Burcelin R., Luche E., Serino M., Amar J. (2009). The gut microbiota ecology: a new opportunity for the treatment of metabolic diseases?. Front. Biosci. (Landmark Ed.).

[bb0025] Cani P.D., Amar J., Iglesias M.A., Poggi M., Knauf C., Bastelica D., Neyrinck A.M., Fava F., Tuohy K.M., Chabo C., Waget A., Delmee E., Cousin B., Sulpice T., Chamontin B., Ferrieres J., Tanti J.F., Gibson G.R., Casteilla L., Delzenne N.M., Alessi M.C., Burcelin R. (2007). Metabolic endotoxemia initiates obesity and insulin resistance. Diabetes.

[bb0030] El Asmar R., Panigrahi P., Bamford P., Berti I., Not T., Coppa G.V., Catassi C., Fasano A. (2002). Host-dependent zonulin secretion causes the impairment of the small intestine barrier function after bacterial exposure. Gastroenterology.

[bb0035] Fasano A., Not T., Wang W., Uzzau S., Berti I., Tommasini A., Goldblum S.E. (2000). Zonulin, a newly discovered modulator of intestinal permeability, and its expression in coeliac disease. Lancet.

[bb0040] Garidou L., Pomie C., Klopp P., Waget A., Charpentier J., Aloulou M., Giry A., Serino M., Stenman L., Lahtinen S., Dray C., Iacovoni J.S., Courtney M., Collet X., Amar J., Servant F., Lelouvier B., Valet P., Eberl G., Fazilleau N., Douin-Echinard V., Heymes C., Burcelin R. (2015). The gut microbiota regulates intestinal CD4 T cells expressing ROR-gamma-t and controls metabolic disease. Cell Metab..

[bb0045] Gibson G.R., Roberfroid M.B. (1995). Dietary modulation of the human colonic microbiota: introducing the concept of prebiotics. J. Nutr..

[bb0050] Gutsmann T., Howe J., Zahringer U., Garidel P., Schromm A.B., Koch M.H., Fujimoto Y., Fukase K., Moriyon I., Martinez-De-Tejada G., Brandenburg K. (2010). Structural prerequisites for endotoxic activity in the Limulus test as compared to cytokine production in mononuclear cells. Innate Immun..

[bb0055] Hill C., Guarner F., Reid G., Gibson G.R., Merenstein D.J., Pot B., Morelli L., Canani R.B., Flint H.J., Salminen S., Calder P.C., Sanders M.E. (2014). Expert consensus document. The International Scientific Association for Probiotics and Prebiotics consensus statement on the scope and appropriate use of the term probiotic. Nat. Rev. Gastroenterol. Hepatol..

[bb0060] Ibarra A., Astbury N.M., Olli K., Alhoniemi E., Tiihonen K. (2015). Effects of polydextrose on different levels of energy intake. A systematic review and meta-analysis. Appetite.

[bb0065] Jie Z., Bang-Yao L., Ming-Jie X., Hai-Wei L., Zu-Kang Z., Ting-Song W., Craig S.A. (2000). Studies on the effects of polydextrose intake on physiologic functions in Chinese people. Am. J. Clin. Nutr..

[bb0070] Kadooka Y., Sato M., Imaizumi K., Ogawa A., Ikuyama K., Akai Y., Okano M., Kagoshima M., Tsuchida T. (2010). Regulation of abdominal adiposity by probiotics (*Lactobacillus gasseri* SBT2055) in adults with obese tendencies in a randomized controlled trial. Eur. J. Clin. Nutr..

[bb0075] Kadooka Y., Sato M., Ogawa A., Miyoshi M., Uenishi H., Ogawa H., Ikuyama K., Kagoshima M., Tsuchida T. (2013). Effect of *Lactobacillus gasseri* SBT2055 in fermented milk on abdominal adiposity in adults in a randomised controlled trial. Br. J. Nutr..

[bb0080] Kallio K.A., Hatonen K.A., Lehto M., Salomaa V., Mannisto S., Pussinen P.J. (2015). Endotoxemia, nutrition, and cardiometabolic disorders. Acta Diabetol..

[bb0085] Klein A., Friedrich U., Vogelsang H., Jahreis G. (2008). *Lactobacillus acidophilus* 74-2 and *Bifidobacterium animalis* subsp lactis DGCC 420 modulate unspecific cellular immune response in healthy adults. Eur. J. Clin. Nutr..

[bb0090] Kok R.G., De Waal A., Schut F., Welling G.W., Weenk G., Hellingwerf K.J. (1996). Specific detection and analysis of a probiotic Bifidobacterium strain in infant feces. Appl. Environ. Microbiol..

[bb0095] Kujala U.M., Kaprio J., Sarna S., Koskenvuo M. (1998). Relationship of leisure-time physical activity and mortality: the Finnish twin cohort. JAMA.

[bb0100] Leber B., Tripolt N.J., Blattl D., Eder M., Wascher T.C., Pieber T.R., Stauber R., Sourij H., Oettl K., Stadlbauer V. (2012). The influence of probiotic supplementation on gut permeability in patients with metabolic syndrome: an open label, randomized pilot study. Eur. J. Clin. Nutr..

[bb0105] Ley R.E., Turnbaugh P.J., Klein S., Gordon J.I. (2006). Microbial ecology: human gut microbes associated with obesity. Nature.

[bb0110] Li S., Guerin-Deremaux L., Pochat M., Wils D., Reifer C., Miller L.E. (2010). NUTRIOSE dietary fiber supplementation improves insulin resistance and determinants of metabolic syndrome in overweight men: a double-blind, randomized, placebo-controlled study. Appl. Physiol. Nutr. Metab..

[bb0115] Liu Z.H., Huang M.J., Zhang X.W., Wang L., Huang N.Q., Peng H., Lan P., Peng J.S., Yang Z., Xia Y., Liu W.J., Yang J., Qin H.L., Wang J.P. (2013). The effects of perioperative probiotic treatment on serum zonulin concentration and subsequent postoperative infectious complications after colorectal cancer surgery: a double-center and double-blind randomized clinical trial. Am. J. Clin. Nutr..

[bb0120] Loman T., Lallukka T., Laaksonen M., Rahkonen O., Lahelma E. (2013). Multiple socioeconomic determinants of weight gain: the Helsinki health study. BMC Public Health.

[bb0125] Minami J., Kondo S., Yanagisawa N., Odamaki T., Xiao J.Z., Abe F., Nakajima S., Hamamoto Y., Saitoh S., Shimoda T. (2015). Oral administration of *Bifidobacterium breve* B-3 modifies metabolic functions in adults with obese tendencies in a randomised controlled trial. J. Nutr. Sci..

[bb0130] Moreno-Navarrete J.M., Sabater M., Ortega F., Ricart W., Fernandez-Real J.M. (2012). Circulating zonulin, a marker of intestinal permeability, is increased in association with obesity-associated insulin resistance. PLoS One.

[bb0135] O'neil P.M., Smith S.R., Weissman N.J., Fidler M.C., Sanchez M., Zhang J., Raether B., Anderson C.M., Shanahan W.R. (2012). Randomized placebo-controlled clinical trial of lorcaserin for weight loss in type 2 diabetes mellitus: the BLOOM-DM study. Obesity (Silver Spring).

[bb0140] Osterberg K.L., Boutagy N.E., Mcmillan R.P., Stevens J.R., Frisard M.I., Kavanaugh J.W., Davy B.M., Davy K.P., Hulver M.W. (2015). Probiotic supplementation attenuates increases in body mass and fat mass during high-fat diet in healthy young adults. Obesity (Silver Spring).

[bb0145] Pacifico L., Bonci E., Marandola L., Romaggioli S., Bascetta S., Chiesa C. (2014). Increased circulating zonulin in children with biopsy-proven nonalcoholic fatty liver disease. World J. Gastroenterol..

[bb0150] Parnell J.A., Reimer R.A. (2009). Weight loss during oligofructose supplementation is associated with decreased ghrelin and increased peptide YY in overweight and obese adults. Am. J. Clin. Nutr..

[bb0155] Pendyala S., Walker J.M., Holt P.R. (2012). A high-fat diet is associated with endotoxemia that originates from the gut. Gastroenterology.

[bb0160] Pi-Sunyer X., Astrup A., Fujioka K., Greenway F., Halpern A., KREMPF M., Lau D.C., Le Roux C.W., Violante Ortiz R., Jensen C.B., Wilding J.P., Obesity, S. & Prediabetes, NNSG (2015). A randomized, controlled trial of 3.0 mg of Liraglutide in weight management. N. Engl. J. Med..

[bb0165] Probert H.M., Apajalahti J.H., Rautonen N., Stowell J., Gibson G.R. (2004). Polydextrose, lactitol, and fructo-oligosaccharide fermentation by colonic bacteria in a three-stage continuous culture system. Appl. Environ. Microbiol..

[bb0170] Putaala H., Salusjarvi T., Nordstrom M., Saarinen M., Ouwehand A.C., Bech Hansen E., Rautonen N. (2008). Effect of four probiotic strains and *Escherichia coli* O157:H7 on tight junction integrity and cyclooxygenase expression. Res. Microbiol..

[bb0175] Roessler A., Friedrich U., Vogelsang H., Bauer A., Kaatz M., Hipler U.C., Schmidt I., Jahreis G. (2008). The immune system in healthy adults and patients with atopic dermatitis seems to be affected differently by a probiotic intervention. Clin. Exp. Allergy.

[bb0180] Russo F., Linsalata M., Clemente C., Chiloiro M., Orlando A., Marconi E., Chimienti G., Riezzo G. (2012). Inulin-enriched pasta improves intestinal permeability and modifies the circulating levels of zonulin and glucagon-like peptide 2 in healthy young volunteers. Nutr. Res..

[bb0185] Sanchez M., Darimont C., Drapeau V., Emady-Azar S., Lepage M., Rezzonico E., Ngom-Bru C., Berger B., Philippe L., Ammon-Zuffrey C., Leone P., Chevrier G., St-Amand E., Marette A., Dore J., Tremblay A. (2014). Effect of *Lactobacillus rhamnosus* CGMCC1.3724 supplementation on weight loss and maintenance in obese men and women. Br. J. Nutr..

[bb0190] Sapone A., De Magistris L., Pietzak M., Clemente M.G., Tripathi A., Cucca F., Lampis R., Kryszak D., Carteni M., Generoso M., Iafusco D., Prisco F., Laghi F., Riegler G., Carratu R., Counts D., Fasano A. (2006). Zonulin upregulation is associated with increased gut permeability in subjects with type 1 diabetes and their relatives. Diabetes.

[bb0195] Stenman L.K., Holma R., Korpela R. (2012). High-fat-induced intestinal permeability dysfunction associated with altered fecal bile acids. World J. Gastroenterol..

[bb0200] Stenman L.K., Waget A., Garret C., Klopp P., Burcelin R., Lahtinen S. (2014). Potential probiotic *Bifidobacterium animalis* ssp. lactis 420 prevents weight gain and glucose intolerance in diet-induced obese mice. Benefic. Microbes.

[bb0205] Stenman L.K., Burcelin R., Lahtinen S. (2015). Establishing a causal link between gut microbes, body weight gain and glucose metabolism in humans—towards treatment with probiotics. Benefic. Microbes.

[bb0210] Stenman L.K., Waget A., Garret C., Briand F., Burcelin R., Sulpice T., Lahtinen S. (2015). Probiotic B420 and prebiotic polydextrose improve efficacy of antidiabetic drugs in mice. Diabetol. Metab. Syndr..

[bb0215] Teixeira T.F., Souza N.C., Chiarello P.G., Franceschini S.C., Bressan J., Ferreira C.L., Peluzio Mdo C. (2012). Intestinal permeability parameters in obese patients are correlated with metabolic syndrome risk factors. Clin. Nutr..

[bb0220] Torgerson J.S., Hauptman J., Boldrin M.N., Sjostrom L. (2004). XENical in the prevention of diabetes in obese subjects (XENDOS) study: a randomized study of orlistat as an adjunct to lifestyle changes for the prevention of type 2 diabetes in obese patients. Diabetes Care.

[bb0225] Tuomilehto J., Lindstrom J., Eriksson J.G., Valle T.T., Hamalainen H., Ilanne-Parikka P., Keinanen-Kiukaanniemi S., Laakso M., Louheranta A., Rastas M., Salminen V., Uusitupa M., Finnish Diabetes Prevention Study, G (2001). Prevention of type 2 diabetes mellitus by changes in lifestyle among subjects with impaired glucose tolerance. N. Engl. J. Med..

[bb0230] Turnbaugh P.J., Ley R.E., Mahowald M.A., Magrini V., Mardis E.R., Gordon J.I. (2006). An obesity-associated gut microbiome with increased capacity for energy harvest. Nature.

[bb0235] Wang W., Uzzau S., Goldblum S.E., Fasano A. (2000). Human zonulin, a potential modulator of intestinal tight junctions. J. Cell Sci..

[bb0240] Zhang D., Zhang L., Zheng Y., Yue F., Russell R.D., Zeng Y. (2014). Circulating zonulin levels in newly diagnosed Chinese type 2 diabetes patients. Diabetes Res. Clin. Pract..

[bb0245] Zunszain P.A., Anacker C., Cattaneo A., Carvalho L.A., Pariante C.M. (2011). Glucocorticoids, cytokines and brain abnormalities in depression. Prog. Neuro-Psychopharmacol. Biol. Psychiatry.

