# Supplementary material for: Probiotic With or Without Fiber Controls Body Fat Mass, Associated With Serum Zonulin, in Overweight and Obese Adults—Randomized Controlled Trial
Source: eBioMedicine. 2016 Oct 26;13:190–200. doi: 10.1016/j.ebiom.2016.10.036 (PMC5264483; doi:10.1016/j.ebiom.2016.10.036)
Supplement: Supplementary file 1 — Supplementary material [file mmc1.pdf]

Supplemental material

a)

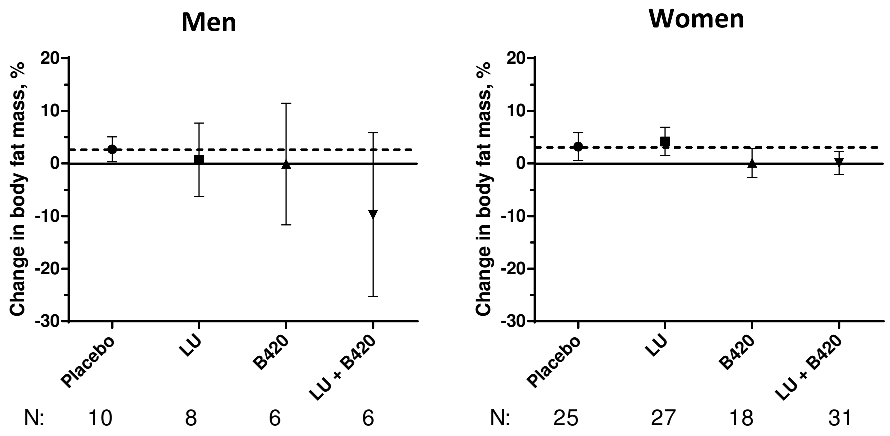

b)

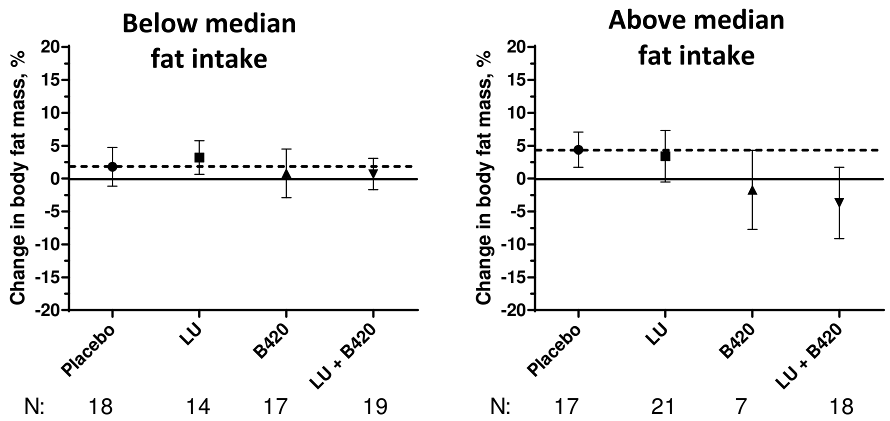

c)

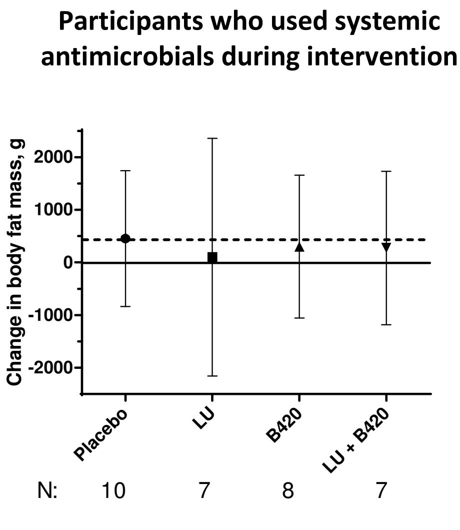

Figure S1. Changes in total body fat mass from baseline to end-of study per sub-group: a) men vs. women, b) below median vs. above median baseline dietary fat intake, and c) those in the Intention-to-Treat population who used systemic antimicrobials during the study. Statistical comparisons were not made due to the low number of participants per group. Data shown as mean +/- 95% Confidence Interval.

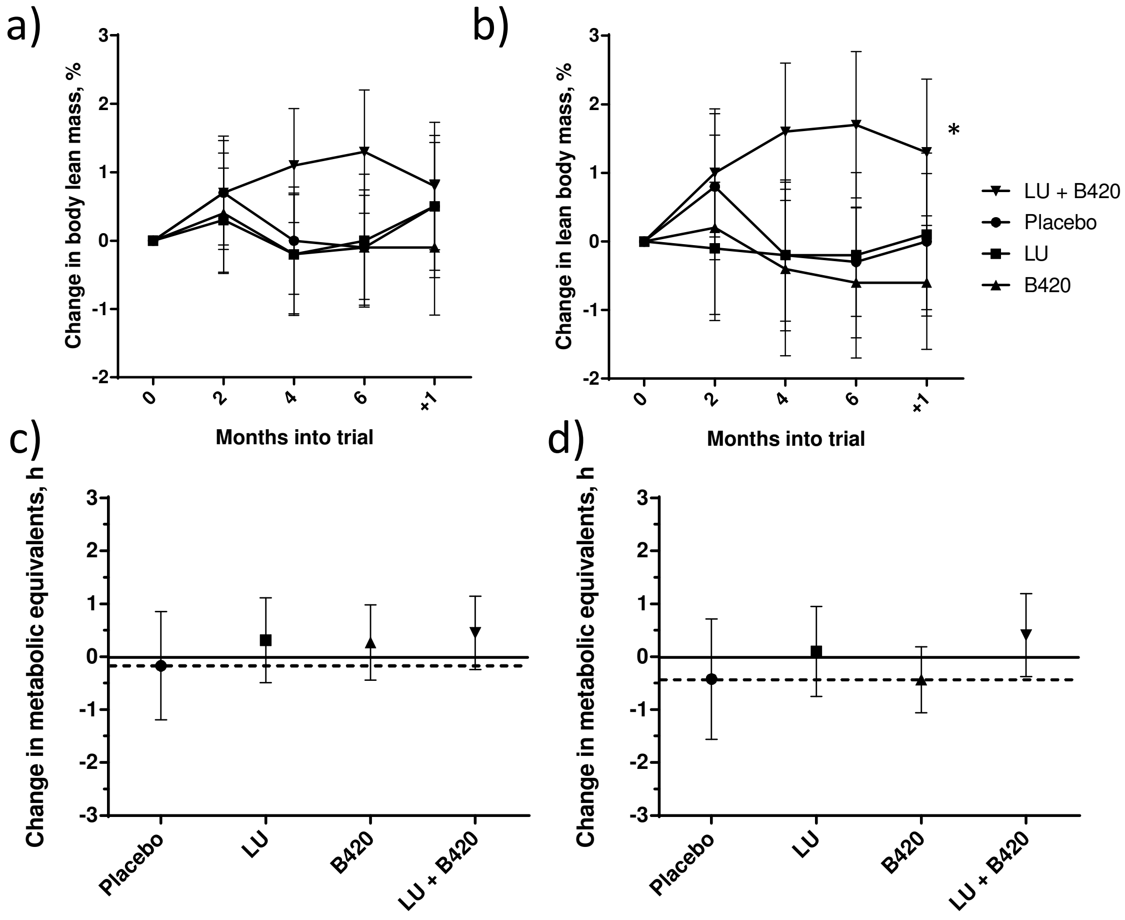

Figure S2. Relative evolution of lean body mass during the study in the ITT (a) and PP populations (b), and questionnaire-reported changes in exercising habits from baseline to end-of-intervention in ITT (c) and PP (d). *P<0.05 for comparison between LU+B420 and Placebo. Overall ANCOVA: a) P = 0.28, b) P = 0.30. Data shown as mean +/- 95% Confidence Interval.

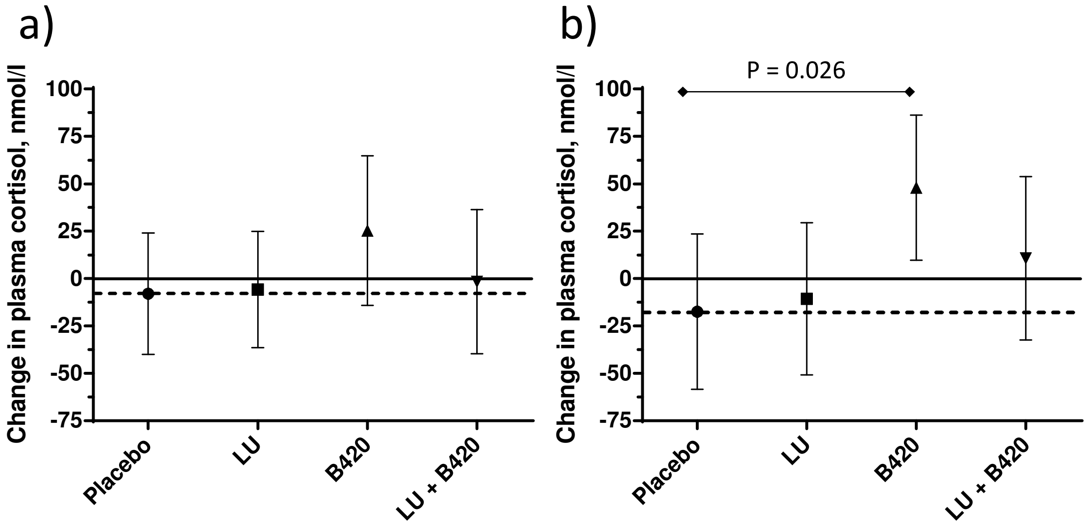

Figure S3. Changes in plasma cortisol concentration from baseline to end-of-intervention in the ITT (a) and PP (b) populations. Study visits were scheduled within a two-hour time window in the morning in order to measure cortisol from the same point of the circadian rythm at each visit. Overall ANCOVA: a) P = 0.36, b) P = 0.060. Data shown as mean +/- 95% Confidence Interval.

Table S1. Analysis of B420 by qPCR in fecal samples at the beginning and end of the study.

	**Samples positive at baseline**[A]	**Average quantity in positive samples at baseline**[B] **[Log^{genome}/ g feces]**	**Samples positive at end-of-study**	**Average quantity in positive samples at end-of-study [Log^{genome}/ g feces]**	**Increased fecal B420 level**[C]
Placebo	21 / 50	8.59 ± 0.92	16 / 50	8.15 ± 0.94	4 / 50
LU	15 / 48	8.16 ± 0.83	17 / 48	8.41 ± 1.02	7 / 48
B420	17 / 38	8.17 ± 0.87	34 / 38	9.24 ± 0.56	32 / 38
LU+B420	17 / 47	8.48 ± 0.87	43 / 47	9.11 ± 0.58	38 / 47

[A]Fecal samples were analysed from all participants who had returned both baseline and end-of-study samples. [B]Limit of detection for the B420 qPCR was 6.40 Log^{genome}/g feces. [C]The samples at 6 months were compared to baseline samples, and participants were considered positive for compliance when the B420 concentration was at least one log higher at the 6-month visit compared to baseline. Participants were considered test negative when the B420 concentration was undetectable at 6 months or when the concentration remained within one log compared to baseline.

Table S2. Weight-related outcomes in the Intention-to-Treat population.

Outcome		**Placebo** *Mean±SD*	**LU** *Mean±SD*	**B420** *Mean±SD*	**LU + B420** *Mean±SD*	*Overall P*	*Factorial P B420*	*Factorial P LU*
	n	44-56	47-53	36-48	41-52			
Total body fat mass (kg) †	*Baseline*	36.8 ± 6.7	36.4 ± 5.4	36.6 ± 5.3	37.6 ± 6.3			
	Month 6[A]	37.8 ± 7.2	37.8 ± 6.3	36.9 ± 5.7	37.8 ± 7.5			
	Δ (%)	+2.5 ± 5.4	+3.2 ± 7.0	+1.3 ± 6.9	-0.9 ± 7.8	0.46	0.012	0.95
Trunk fat mass (kg) †	*Baseline*	19.6 ± 3.8	19.5 ± 2.9	19.9 ± 3.1	19.9 ± 3.4			
	Month 6	20.3 ± 4.0	20.4 ± 3.6	20.4 ± 3.6	20.0 ± 4.3			
	Δ (%)	+3.8 ± 7.0	+4.7 ± 10.9	+1.9 ± 9.0	-0.8 ± 9.6	0.43	0.012	0.97
Total lean body mass (kg)	*Baseline*	47.9 ± 9.2	49.3 ± 9.7	48.5 ± 9.1	46.6 ± 9.3			
	Month 6	48.8 ± 9.6	49.2 ± 9.5	49.1 ± 9.7	46.7 ± 9.3			
	Δ (%)	-0.1 ± 2.5	0.0 ± 3.3	-0.1 ± 2.6	+1.3 ± 3.1	0.28	0.064	0.16
Body weight (kg)	*Baseline*	88.5 ± 12.2	89.4 ± 9.05	88.7 ± 9.25	87.8 ± 11.0			
	Month 6	89.8 ± 12.6	90.5 ± 9.86	89.6 ± 10.4	87.8 ± 11.8			
	Δ (%)	+1.0 ± 2.8	+0.9 ± 3.1	+0.4 ± 3.1	+0.2 ± 3.1	0.47	0.12	0.92
Waist circumference (cm) †	*Baseline*	103.0 ± 8.4	103.4 ± 6.1	102.6 ± 6.9	102.5 ± 6.9			
	Month 6	103.6 ± 8.5	104.0 ± 7.5	102.8 ± 6.9	100.3 ± 8.6			
	Δ (%)	+0.6 ± 5.0	+0.5 ± 4.4	+0.2 ± 5.2	-2.0 ± 4.5*	0.17	0.023	0.10
Energy intake (kcal/day)[B]	*Baseline*	2140 ± 570	2210 ± 590	2170 ± 440	2080 ± 630			
	Month 6	2120 ± 450	2050 ± 520	2040 ± 550	1900 ± 470			
	*Δ (*kcal/d*)*	-4.4 ± 540	-161 ± 470	-190 ± 470	-124 ± 630	0.23	0.50	0.11

[A]=Mean values of each time point and differences contain only measured values. For statistical analyses, last observation was carried forward to account for missing values [B]Women with energy intake < 80% and men with energy intake < 85% of basal metabolic rate excluded from dietary intake analyses. The energy content of the smoothie vehicle is included in the results.

* = Significant difference from Placebo, P < 0.05 (Dunnett's test, corrected for multiple comparisons, last observation carried forward for missing observations). Only relative changes from baseline to month 6 were compared statistically between groups. †=Factorial analysis for B420, P < 0.05.

Table S3. Distribution of the most common adverse events (AE) in all 225 randomized participants and changes in safety parameters from baseline to 6 months in the ITT and PP populations.

	**Placebo**	**Lit. Ultra**	**B420**	**LU + B420**	**Total**
	n of cases	*n of cases*	*n of cases*	*n of cases*	*n of cases*
Any AE	48	50	52	49	199
Nasopharyngitis	16	12	16	16	60
Headache	8	10	10	17	45
Diarrhea (incl. loose stools)	6	6	9	11	32
Influenza	7	5	8	10	30
Flatulence	4	11	2	8	25
Back pain	5	6	5	5	21

	**Placebo**	**Lit. Ultra**	**B420**	**LU + B420**	Overall P
Outcome	*Δ mean±SD*	*Δ mean±SD*	*Δ mean±SD*	*Δ mean±SD*	
**Intention-to-Treat**					
Blood pressure, systolic (mmHg)	-2.1 ± 13.2	-1.8 ± 11.7	-1.2 ± 10.7	-2.9 ± 12.3	0.73
Blood pressure, diastolic (mmHg)	-0.7 ± 7.1	-1.5 ± 8.3	-0.3 ± 7.7	-2.9 ± 7.5	0.22
Heart rate (beats / min)	+0.6 ± 7.5	-0.7 ± 7.4	+0.7 ± 9.2	-0.5 ± 8.8	0.68
ASAT (U/l)	+0.8 ± 7.9	+0.5 ± 9.4	-0.1 ± 7.3	+0.1 ± 6.8	0.56
ALAT (U/l)	+0.9 ± 19.2	-2.2 ± 10.9	-2.0 ± 12.6	-2.3 ± 9.0	0.16
gamma-GT (U/l)	+1.2 ± 10.3	+3.2 ± 31.4	-1.4 ± 27.0	-1.8 ± 24.7	0.57
**Per Protocol**					
Blood pressure, systolic (mmHg)	-3.1 ± 11.4	-3.5 ± 13.0	-1.8 ± 11.9	-2.3 ± 12.1	0.47
Blood pressure, diastolic (mmHg)	-1.0 ± 6.6	-4.1 ± 7.5	-0.8 ± 7.8	-2.4 ± 7.6	0.10
Heart rate (beats / min)	-0.8 ± 7.4	-0.6 ± 7.7	+1.0 ± 9.4	-0.9 ± 8.9	0.43
ASAT (U/l)	+1.0 ± 8.8	+1.4 ± 8.5	-0.0 ± 7.0	+0.2 ± 6.1	0.90
ALAT (U/l)	+1.3 ± 21.9	-0.9 ± 10.9	-1.2 ± 13.4	-1.8 ± 9.7	0.36
gamma-GT (U/l)	+1.7 ± 11.6	+6.4 ± 32.9	-3.9 ± 33.5	-2.6 ± 28.1	0.81

Table S4. Body fat mass in different regions of the body (Per Protocol population).

Outcome		**Placebo** *Mean±SD*	**LU** *Mean±SD*	**B420** *Mean±SD*	**LU + B420** *Mean±SD*	*Overall P*	*Factorial P B420*	*Factorial P LU*
	n	35	35	24	37			
Total body fat mass (kg)	*Baseline*	36.4 ± 6.8	36.6 ± 5.0	35.9 ± 5.2	37.9 ± 6.8			
	Month 6	37.5 ± 7.1	37.9 ± 6.2	35.9 ± 5.7	37.6 ± 8.1			
	Δ (%)	+3.1 ± 5.6	+3.4 ± 7.2	+0.1 ± 7.0	-1.5 ± 8.5*	0.095	0.002	0.67
Trunk fat mass (kg)	*Baseline*	19.3 ± 3.8	19.5 ± 2.8	19.7 ± 3.7	20.2 ± 3.6			
	Month 6	20.2 ± 4.0	20.5 ± 3.8	19.6 ± 3.7	20.0 ± 4.6			
	Δ (%)	+5.0 ± 6.7	+5.3 ± 11.4	-0.4 ± 8.5	-1.7 ± 10.1*	0.036	0.0002	0.79
Arm fat mass (kg)	*Baseline*	3.97 ± 1.0	4.06 ± 1.0	3.96 ± 1.2	4.33 ± 1.0			
	Month 6	3.92 ± 0.77	3.96 ± 0.84	3.91 ± 1.3	4.18 ± 1.0			
	Δ (%)	+1.1 ± 15.8	+0.1 ± 17.1	-0.6 ± 15.8	-2.6 ± 14.2	0.67	0.62	0.98
Leg fat mass (kg)	*Baseline*	12.2 ± 3.9	12.0 ± 3.2	11.2 ± 2.6	12.4 ± 3.3			
	Month 6	12.4 ± 4.1	12.4 ± 3.5	11.4 ± 2.8	12.5 ± 3.6			
	Δ (%)	+1.5 ± 7.4	+2.8 ± 8.2	+1.7 ± 8.2	+0.1 ± 9.5	0.98	0.34	0.99
Android fat mass (kg)	*Baseline*	3.38 ± 0.74	3.43 ± 0.71	3.51 ± 0.82	3.51 ± 0.78			
	Month 6	3.55 ± 0.84	3.67 ± 0.91	3.48 ± 0.82	3.55 ± 0.96			
	Δ (%)	+5.2 ± 11.0	+6.9 ± 13.7	-0.4 ± 9.0	+0.5 ± 11.9	0.23	0.004	0.50
Gynoid fat mass (kg)	*Baseline*	6.18 ± 1.6	5.95 ± 1.2	5.92 ± 1.1	6.12 ± 1.5			
	Month 6	6.37 ± 1.7	6.29 ± 1.4	5.99 ± 1.2	6.22 ± 1.7			
	Δ (%)	2.9 ± 7.2	5.9 ± 12.1	1.3 ± 8.0	1.3 ± 10.5	0.98	0.064	0.34

* = Significant difference from Placebo, P < 0.05 (Dunnett's test, corrected for multiple comparisons). Only relative changes from baseline to month 6 were statistically compared between groups.

Table S5. Markers of glucose metabolism in the Intention-to-Treat and Per Protocol populations

Outcome		**Placebo** *Mean±SD*	**LU** *Mean±SD*	**B420** *Mean±SD*	**LU + B420** *Mean±SD*	*Overall P*	*Factorial P B420*	*Factorial P LU*
**Intention-to-Treat**	*n*	49-56	51-53	42-48	48-52			
Glucose (mmol/l)	*Baseline*	5.24 ± 0.50	5.20 ± 0.62	5.18 ± 0.43	5.19 ± 0.42			
	Month 6	5.31 ± 0.51	5.32 ± 0.51	5.23 ± 0.43	5.35 ± 0.49			
	Δ	+0.04 ± 0.32	+0.11 ± 0.49	+0.06 ± 0.30	+0.13 ± 0.38	0.34	0.71	0.21
Insulin (mU/l)	*Baseline*	8.29 ± 4.5	7.84 ± 3.7	9.07 ± 5.0	8.43 ± 6.3			
	Month 6	7.49 ± 4.0	7.84 ± 4.0	7.90 ± 3.9	7.91 ± 4.5			
	Δ	-0.30 ± 3.0	+0.09 ± 3.2	-1.20 ± 4.0	-0.87 ± 5.7	0.92	0.56	0.85
HOMA-IR (arbitrary unit)	*Baseline*	1.97 ± 1.2	1.86 ± 1.0	2.11 ± 1.2	1.98 ± 1.5			
	Month 6	1.81 ± 1.2	1.89 ± 1.1	1.87 ± 1.0	1.93 ± 1.2			
	Δ	-0.05 ± 0.76	+0.05 ± 0.84	-0.24 ± 1.0	-0.13 ± 1.4	0.85	0.67	0.73
HbA1c (mmol/mol)	*Baseline*	35.0 ± 3.2	34.8 ± 3.7	35.3 ± 3.4	34.7 ± 3.6			
	Month 6	35.4 ± 3.8	35.5 ± 4.3	35.2 ± 3.5	35.1 ± 3.5			
	Δ	+0.4 ± 3.3	+0.6 ± 3.3	-0.0 ± 3.0	+0.3 ± 2.4	0.92	0.31	0.44

Outcome		**Placebo** *Mean±SD*	**LU** *Mean±SD*	**B420** *Mean±SD*	**LU + B420** *Mean±SD*	*Overall P*	*Factorial P B420*	*Factorial P LU*
**Per Protocol**	*n*	36	36	25	37			
Glucose (mmol/l)	*Baseline*	5.21 ± 0.54	5.18 ± 0.68	5.16 ± 0.35	5.22 ± 0.42			
	Month 6	5.24 ± 0.49	5.28 ± 0.54	5.22 ± 0.43	5.43 ± 0.48			
	Δ	+0.02 ± 0.33	+0.10 ± 0.54	+0.06 ± 0.33	+0.20 ± 0.36	0.21	0.26	0.072
Insulin (mU/l)	*Baseline*	8.09 ± 4.3	8.44 ± 3.8	9.15 ± 6.1	8.15 ± 4.3			
	Month 6	7.41 ± 3.8	8.37 ± 4.2	7.24 ± 3.8	8.27 ± 4.7			
	Δ	-0.54 ± 3.0	-0.07 ± 3.5	-1.92 ± 4.5	+0.12 ± 4.1	0.60	0.61	0.035
HOMA-IR (arbitrary unit)	*Baseline*	1.93 ± 1.2	2.00 ± 1.1	2.10 ± 1.4	1.91 ± 1.1			
	Month 6	1.77 ± 1.1	2.02 ± 1.2	1.70 ± 1.0	2.04 ± 1.3			
	Δ	-0.12 ± 0.74	+0.01 ± 0.94	-0.40 ± 1.1	+0.13 ± 1.0	0.53	0.86	0.035
HbA1c (mmol/mol)	*Baseline*	34.7 ± 2.8	34.6 ± 3.7	35.7 ± 3.5	35.2 ± 3.3			
	Month 6	34.7 ± 3.5	35.7 ± 4.8	35.6 ± 3.9	35.5 ± 3.4			
	Δ	+0.0 ± 3.1	+1.0 ± 3.4	-0.1 ± 3.5	+0.2 ± 2.6	0.40	0.60	0.22

Table S6. Cardiovascular biomarkers in the Intention-to-Treat and Per Protocol populations

Outcome		Placebo *Mean±SD*	LU *Mean±SD*	B420 *Mean±SD*	LU + B420 *Mean±SD*	*Overall P*	*Factorial P B420*	*Factorial P LU*
**Intention-to-Treat**	*n*	47-56	49-53	40-48	48-52			
Total cholesterol (mmol/l)	*Baseline*	5.23 ± 0.87	5.22 ± 1.00	5.26 ± 1.08	5.50 ± 0.96			
	Month 6	5.24 ± 1.02	5.23 ± 0.90	5.25 ± 0.98	5.43 ± 0.88			
	Δ	+0.05 ± 0.65	-0.01 ± 0.53	+0.07 ± 0.54	-0.09 ± 0.54	0.96	0.82	0.60
LDL cholesterol (mmol/l)	*Baseline*	3.10 ± 0.77	3.08 ± 0.82	3.25 ± 0.91	3.38 ± 0.89			
	Month 6	3.13 ± 0.84	3.05 ± 0.71	3.20 ± 0.77	3.30 ± 0.73			
	Δ	+0.04 ± 0.48	-0.05 ± 0.51	+0.03 ± 0.46	-0.08 ± 0.44	0.73	0.70	0.54
HDL cholesterol (mmol/l)	*Baseline*	1.57 ± 0.44	1.56 ± 0.40	1.42 ± 0.34	1.54 ± 0.36			
	Month 6	1.58 ± 0.46	1.57 ± 0.38	1.39 ± 0.31	1.51 ± 0.34			
	Δ	+0.01 ± 0.22	+0.02 ± 0.21	-0.04 ± 0.19	-0.04 ± 0.25	0.18	0.032	0.92
Triglycerides (mmol/l)	*Baseline*	1.22 ± 0.61	1.27 ± 0.53	1.28 ± 0.56	1.30 ± 0.62			
	Month 6	1.19 ± 0.50	1.32 ± 0.78	1.47 ± 0.69	1.39 ± 0.82			
	Δ	+0.01 ± 0.40	+0.06 ± 0.57	+0.19 ± 0.52*	+0.09 ± 0.58	0.049	0.042	0.98
oxLDL (U/l)	*Baseline*	51.5 ± 12.7	51.1 ± 12.4	53.2 ± 12.7	56.4 ± 12.8			
	Month 6	52.8 ± 14.3	53.4 ± 12.9	55.5 ± 13.7	58.4 ± 13.0			
	Δ (U/l)	+1.59 ± 9.77	+2.27 ± 7.14	+2.58 ± 7.30	+2.00 ± 7.65	0.46	0.52	0.70
ICAM-1 (ng/ml)	*Baseline*	334 ± 83	328 ± 86	346 ± 93	329 ± 77			
	Month 6	348 ± 90	328 ± 78	366 ± 121	330 ± 89			
	Δ (ng/ml)	+18 ± 77	-0 ± 57	+22 ± 77	+1 ± 68	0.38	0.74	0.039
VCAM-1 (ng/ml)	*Baseline*	1530 ± 586	1450 ± 438	1560 ± 402	1420 ± 349			
	Month 6	1500 ± 515	1400 ± 413	1620 ± 426	1380 ± 388			
	Δ (ng/ml)	-12 ± 405	-55 ± 200	+55 ± 325	-40 ± 282	0.88	0.28	0.024
PAI-1 (ng/ml)	*Baseline*	56 ± 30	68 ± 58	65 ± 36	72 ± 64			
	Month 6	56 ± 27	54 ± 28	61 ± 34	57 ± 33			
	Δ (ng/ml)	+1 ± 28	-14 ± 56	-3 ± 45	-15 ± 66	0.61[A]	0.95[A]	0.29[A]
E-selectin (ng/ml)	*Baseline*	22.8 ± 8.14	23.3 ± 7.02	23.1 ± 9.62	22.1 ± 8.11			
	Month 6	23.0 ± 7.23	24.61 ± 8.22	24.1 ± 10.46	22.8 ± 8.59			
	Δ (ng/ml)	+0.86 ± 4.18	+1.44 ± 3.88	+1.62 ± 4.25	+0.83 ± 3.79	0.28	0.95	0.58

Outcome		**Placebo** *Mean±SD*	**LU** *Mean±SD*	**B420** *Mean±SD*	**LU + B420** *Mean±SD*	*Overall P*	*Factorial P B420*	*Factorial P LU*
**Per Protocol**	*n*	36	36	25	37			
Total cholesterol (mmol/l)	*Baseline*	5.16 ± 0.86	5.24 ± 1.01	5.14 ± 0.86	5.58 ± 0.90			
	Month 6	5.27 ± 1.03	5.22 ± 0.87	5.16 ± 0.84	5.52 ± 0.87			
	Δ	+0.11 ± 0.67	-0.03 ± 0.51	+0.02 ± 0.48	-0.06 ± 0.50	0.34	0.79	0.48
LDL cholesterol (mmol/l)	*Baseline*	3.05 ± 0.75	3.12 ± 0.73	3.12 ± 0.66	3.39 ± 0.84			
	Month 6	3.14 ± 0.85	3.08 ± 0.60	3.16 ± 0.64	3.35 ± 0.75			
	Δ	+0.09 ± 0.49	-0.04 ± 0.53	+0.04 ± 0.42	-0.04 ± 0.38	0.43	0.81	0.37
HDL cholesterol (mmol/l)	*Baseline*	1.56 ± 0.45	1.56 ± 0.44	1.41 ± 0.40	1.57 ± 0.39			
	Month 6	1.56 ± 0.50	1.54 ± 0.40	1.34 ± 0.32	1.51 ± 0.33			
	Δ	+0.03 ± 0.25	-0.02 ± 0.20	-0.08 ± 0.19	-0.06 ± 0.26	0.043	0.042	0.93
Triglycerides (mmol/l)	*Baseline*	1.20 ± 0.67	1.25 ± 0.54	1.33 ± 0.58	1.36 ± 0.66			
	Month 6	1.21 ± 0.53	1.32 ± 0.84	1.49 ± 0.66	1.47 ± 0.82			
	Δ	+0.01 ± 0.41	+0.07 ± 0.65	+0.16 ± 0.56	+0.10 ± 0.64	0.25	0.24	0.86
oxLDL (U/l)	*Baseline*	50.2 ± 11.3	51.7 ± 12.2	53.2 ± 10.4	56.8 ± 12.2			
	Month 6	53.1 ± 15.2	53.7 ± 11.2	55.7 ± 13.5	58.9 ± 12.5			
	Δ (U/l)	+2.92 ± 9.32	+1.96 ± 6.52	+2.54 ± 6.63	+2.06 ± 7.93	0.63	0.93	0.58
ICAM-1 (ng/ml)	*Baseline*	318 ± 82	328 ± 95	338 ± 105	319 ± 81			
	Month 6	342 ± 100	328 ± 85	346 ± 142	326 ± 95			
	Δ (ng/ml)	+24 ± 75	+0 ± 62	+8 ± 74	+8 ± 72	0.22	0.85	0.26
VCAM-1 (ng/ml)	*Baseline*	1500 ± 641	1480 ± 471	1620 ± 369	1380 ± 361			
	Month 6	1500 ± 555	1400 ± 425	1600 ± 426	1380 ± 403			
	Δ (ng/ml)	-2 ± 438	-79 ± 193	-19 ± 279	+0 ± 269	0.57	0.46	0.20
PAI-1 (ng/ml)	*Baseline*	56 ± 29	76 ± 62	61 ± 42	71 ± 68			
	Month 6	59 ± 29	58 ± 29	61 ± 41	57 ± 33			
	Δ (ng/ml)	+3 ± 30	-18 ± 63	-0 ± 54	-14 ± 74	0.42[A]	0.57[A]	0.46[A]
E-selectin (ng/ml)	*Baseline*	20.9 ± 7.01	22.9 ± 6.91	24.1 ± 10.18	21.7 ± 7.42			
	Month 6	21.9 ± 7.09	24.4 ± 8.22	24.8 ± 11.31	22.6 ± 8.42			
	Δ (ng/ml)	+1.03 ± 4.27	+1.48 ± 3.99	+0.72 ± 4.24	+0.96 ± 3.82	0.92	0.40	0.56

[A]Analyses conducted on log-transformed data

Table S7. Baseline dietary intake in the Intention-to-Treat and Per-Protocol populations.

		Placebo	LU	B420	LU + B420	Total
		Mean±SD	*Mean±SD*	*Mean±SD*	*Mean±SD*	*Mean±SD*
**Intention-to-Treat**[A]	*n*	53	50	45	50	198
Energy, kcal/day		2140 ± 572	2210 ± 593	2170 ± 442	2080 ± 626	2150 ± 563
Protein, %kcal		17.1 ± 2.9	17.6 ± 4.3	17.4 ± 3.8	16.5 ± 3.0	17.1 ± 3.5
Fat, %kcal		37.2 ± 6.3	37.3 ± 5.0	38.3 ± 5.0	37.1 ± 6.2	37.5 ± 5.7
Saturated fatty acids, %kcal		13.9 ± 3.3	13.7 ± 2.8	14.2 ± 3.1	13.7 ± 3.0	13.9 ± 3.0
Monounsaturated fatty acids, %kcal		12.7 ± 2.5	12.5 ± 1.8	12.9 ± 2.1	12.6 ± 3.0	12.7 ± 2.4
Polyunsaturated fatty acids, %kcal		6.0 ± 1.3	6.1 ± 1.3	6.4 ± 2.1	6.0 ± 1.8	6.1 ± 1.6
Carbohydrate, %kcal		39.7 ± 6.7	39.4 ± 5.9	38.7 ± 6.9	40.0 ± 7.2	39.5 ± 6.7
Sucrose, %kcal		8.6 ± 3.3	9.3 ± 3.6	8.9 ± 4.1	9.6 ± 3.9	9.1 ± 3.7
Fiber, g/day		20.6 ± 6.4	20.9 ± 6.9	22.5 ± 7.1	20.7 ± 6.6	21.1 ± 6.7
**Per-Protocol**[A]	*n*	33	33	24	35	132
Energy, kcal/day		2240 ± 512	2210 ± 654	2200 ± 381	2090 ± 643	2180 ± 567
Protein, %kcal		16.6 ± 2.0	17.7 ± 4.8	16.3 ± 2.3	16.0 ± 3.1	16.7 ± 3.3
Fat, %kcal		38.2 ± 7.0	37.5 ± 4.7	38.0 ± 4.8	37.2 ± 6.4	37.7 ± 5.8
Saturated fatty acids, %kcal		14.6 ± 3.6	13.6 ± 2.9	14.4 ± 3.3	14.0 ± 3.1	14.1 ± 3.2
Monounsaturated fatty acids, %kcal		12.9 ± 2.7	12.7 ± 1.7	13.0 ± 2.0	12.6 ± 3.0	12.8 ± 2.4
Polyunsaturated fatty acids, %kcal		5.9 ± 1.4	6.3 ± 1.4	6.0 ± 1.8	5.8 ± 1.7	6.0 ± 1.6
Carbohydrate, %kcal		39.8 ± 6.0	39.7 ± 6.4	40.2 ± 6.4	40.7 ± 7.4	40.1 ± 6.5
Sucrose, %kcal		8.6 ± 3.3	9.9 ± 3.8	9.0 ± 4.6	10.2 ± 4.2	9.4 ± 4.0
Fiber, g/day		21.4 ± 6.6	22.0 ± 7.9	23.7 ± 6.6	20.0 ± 6.2	21.6 ± 6.9

[A]Women with energy intake < 80% and men with energy intake < 85% of basal metabolic rate excluded (n of exclusions 1-2 per group).

Table S8. Markers of endotoxemia and low-grade inflammation (Intention-to-Treat population).

Outcome		**Placebo** *Mean±SD*	**LU** *Mean±SD*	**B420** *Mean±SD*	**LU + B420** *Mean±SD*	*Overall P*	*Factorial P B420*	*Factorial P LU*
	n	46-48	49-50	40-47	48-50			
Zonulin (ng/ml)	*Baseline*	57.0 ± 11	55.3 ± 9.0	59.4 ± 13	63.5 ± 14			
	Month 6	59.4 ± 11	57.9 ± 13	58.2 ± 12	62.4 ± 12			
	Δ (ng/ml)	+2.5 ± 9.6	+2.6 ± 11	-0.62 ± 8.4	-1.1 ± 7.0	0.28	0.11	0.83
ApoB-48 (µg/ml)	*Baseline*	10.5 ± 6.6	10.3 ± 6.6	10.8 ± 5.6	9.3 ± 4.7			
	Month 6	10.3 ± 5.7	9.4 ± 5.1	11.8 ± 6.6	10.1 ± 5.8			
	Δ (µg/ml)	-0.12 ± 5.1	-0.92 ± 5.0	+0.91 ± 5.9	+0.81 ± 4.3	0.94	0.087[A]	0.15[A]
hsCRP (mg/l)	*Baseline*	2.30 ± 2.0	2.28 ± 2.4	2.61 ± 2.4	2.46 ± 2.4			
	Month 6	2.47 ± 2.0	2.43 ± 2.4	2.57 ± 2.8	2.54 ± 2.4			
	Δ (mg/l)	+0.29 ± 1.9	+0.18 ± 1.8	-0.08 ± 2.2	-0.05 ± 1.9	0.49	0.39	0.89
LPS (EU/l)	*Baseline*	64 ± 100	90 ± 170	47 ± 65	35 ± 37			
	Month 6	45 ± 38	84 ± 310	44 ± 62	48 ± 38			
	Δ (EU/l)	-26 ± 100	-6.2 ± 290	+0.7 ± 38	+13 ± 37*	0.005	0.12[A]	0.059[A]
sCD14 (µg/ml)	*Baseline*	1.67 ± 0.45	1.82 ± 0.50	1.82 ± 0.41	1.75 ± 0.45			
	Month 6	1.79 ± 0.58	1.88 ± 0.49	1.86 ± 0.49	1.77 ± 0.46			
	Δ (µg/ml)	+0.13 ± 0.6	+0.05 ± 0.5	+0.05 ± 0.5	+0.03 ± 0.5	0.33	0.49	0.96
IL-6 (pg/ml)	*Baseline*	10.4 ± 15	82.2 ± 510	9.3 ± 9.8	14.2 ± 46			
	Month 6	11.8 ± 19	107 ± 670	11.0 ± 12	7.9 ± 9.4			
	Δ (pg/ml)	+1.4 ± 16	+11 ± 87	+1.7 ± 11	-7.2 ± 41	0.52	0.28[A]	0.20[A]

* = Significant difference from Placebo, $P < 0.05$ (Dunnett's test, corrected for multiple comparisons). Only relative changes from baseline to month 6 were statistically compared between groups.

[A]Analysis of log transformed values

Table S9. Adipose tissue biomarkers in the Intention-to-Treat and Per Protocol populations.

Outcome		Placebo *Mean±SD*	LU *Mean±SD*	B420 *Mean±SD*	LU + B420 *Mean±SD*	*Overall P*	*Factorial P B420*	*Factorial P LU*
**Intention-to-Treat**	*n*	49-56	51-53	42-48	48-52			
Leptin (ng/ml)	*Baseline*	34.8 ± 18	34.0 ± 18	35.9 ± 19	39.6 ± 17			
	Month 6	36.2 ± 20	34.3 ± 19	35.6 ± 21	43.7 ± 20			
	Δ (ng/ml)	+2.3 ± 9.3	+0.41 ± 10.8	+0.99 ± 9.9	+2.6 ± 11.8	0.48	0.73	0.73
Adiponectin (µg/ml)	*Baseline*	9.39 ± 5.6	9.98 ± 5.8	7.93 ± 4.4	9.84 ± 5.5			
	Month 6	10.6 ± 8.3	10.3 ± 5.6	7.92 ± 4.9	9.65 ± 5.1			
	Δ (µg/ml)	+0.86 ± 5.0	+0.28 ± 3.5	-0.00 ± 2.0	-0.48 ± 2.9	0.83[A]	0.17[A]	0.86[A]
MCP-1 (pg/ml)	*Baseline*	311 ± 180	314 ± 130	328 ± 150	353 ± 170			
	Month 6	265 ± 95	316 ± 130	367 ± 190	303 ± 130			
	Δ (pg/ml)	-43 ± 160	+6.7 ± 75	+36.5 ± 180*	-43.8 ± 120	0.003	0.049	0.53
Angptl4 (ng/ml)	*Baseline*	189 ± 578	128 ± 186	74.6 ± 43.3	354 ± 1330			
	Month 6	156 ± 394	127 ± 191	75.9 ± 41.7	256 ± 745			
	Δ (ng/ml)	-29.5 ± 212	-0.48 ± 31.0	+1.02 ± 20.1	-98.0 ± 609	0.48[A]	0.50[A]	0.22[A]

Outcome		Placebo *Mean±SD*	LU *Mean±SD*	B420 *Mean±SD*	LU + B420 *Mean±SD*	*Overall P*	*Factorial P B420*	*Factorial P LU*
**Per Protocol**	*n*	36	36	25	36-37			
Leptin (ng/ml)	*Baseline*	32.2 ± 19	33.5 ± 18	32.2 ± 19	41.3 ± 18			
	Month 6	34.9 ± 19	34.2 ± 16	32.9 ± 20	44.4 ± 21			
	Δ (ng/ml)	+2.7 ± 9.9	+0.71 ± 11.3	+0.78 ± 9.5	+3.1 ± 11.7	0.72	0.59	0.77
Adiponectin (µg/ml)	*Baseline*	9.52 ± 6.1	9.53 ± 6.2	7.87 ± 3.7	9.90 ± 5.3			
	Month 6	10.6 ± 9.0	9.67 ± 5.4	7.51 ± 3.5	9.13 ± 4.5			
	Δ (µg/ml)	+1.10 ± 5.6	+0.14 ± 3.0	-0.36 ± 2.2	-0.77 ± 3.2	0.13[A]	0.013[A]	0.91[A]
MCP-1 (pg/ml)	*Baseline*	325 ± 210	314 ± 140	346 ± 190	346 ± 160			
	Month 6	280 ± 94	324 ± 150	388 ± 200	294 ± 130			
	Δ (pg/ml)	-44.5 ± 180	+9.4 ± 84	+41.8 ± 220*	-51.2 ± 120	0.03	0.35	0.46
Angptl4 (ng/ml)	*Baseline*	216 ± 666	132 ± 199	73.2 ± 28.6	421 ± 1520			
	Month 6	179 ± 449	131 ± 27.9	78.6 ± 33.5	302 ± 847			
	Δ (ng/ml)	-36.6 ± 243	-0.66 ± 34.7	+5.37 ± 18.4	-119 ± 695	0.50[A]	0.97[A]	0.11[A]

[A]Analysis of log transformed values

Table S10. Changes in bacterial metabolites in feces in the Intention to Treat population.

		**Placebo** Mean±SD (µmol/g)	**LU** Mean±SD (µmol/g)	**B420** Mean±SD (µmol/g)	**LU + B420** Mean±SD (µmol/g)	*Overall P*	*Factorial P B420*	*Factorial P LU*
	n	47-56	47-53	37-46	45-52			
Acetic acid	*Baseline*	42.3 ± 15	43.6 ± 18	37.7 ± 20	44.0 ± 18			
	Month 6	39.7 ± 18	39.7 ± 15	38.6 ± 14	43.6 ± 19			
	Δ	-3.00 ± 15	-4.58 ± 18	-1.00 ± 24	-1.04 ± 24	0.74	0.42	0.56
Propionic acid	*Baseline*	12.1 ± 5.4	13.4 ± 8.5	10.8 ± 6.0	12.8 ± 6.3			
	Month 6	10.5 ± 5.4	11.4 ± 6.5	11.6 ± 4.8	12.6 ± 5.7			
	Δ	-1.40 ± 4.5	-1.92 ± 6.5	+0.22 ± 6.8	-0.01 ± 7.6	0.17	0.069	0.57
Butyric acid	*Baseline*	11.2 ± 6.8	11.1 ± 6.4	9.83 ± 7.1	11.8 ± 6.5			
	Month 6	10.7 ± 6.2	9.77 ± 5.2	11.9 ± 8.1	11.2 ± 5.6			
	Δ	-0.95 ± 6.0	-1.59 ± 6.4	+1.25 ± 11	-0.79 ± 7.5	0.76	0.14	0.27
Valeric acid	*Baseline*	1.62 ± 1.0	1.58 ± 1.1	1.46 ± 1.0	1.78 ± 0.92			
	Month 6	1.39 ± 0.89	1.47 ± 0.95	1.73 ± 1.2	1.59 ± 0.96			
	Δ	-0.20 ± 1.0	-0.12 ± 1.3	+0.13 ± 1.4	-0.19 ± 1.2	0.21	0.14	0.23
Lactic acid	*Baseline*	0.69 ± 0.80	0.75 ± 0.89	0.68 ± 0.79	0.71 ± 0.77			
	Month 6	0.64 ± 0.72	0.72 ± 0.82	1.1 ± 2.2	0.76 ± 0.82			
	Δ	-0.08 ± 0.83	+0.08 ± 0.82	+0.43 ± 1.8	+0.11 ± 0.86	0.35[B]	0.75[B]	0.78[B]
Branched-chain fatty acids[A]	*Baseline*	3.62 ± 2.1	3.35 ± 2.1	3.56 ± 2.0	3.90 ± 1.9			
	Month 6	2.92 ± 1.3	3.03 ± 1.4	3.77 ± 3.0	3.04 ± 1.9			
	Δ	-0.84 ± 2.4	-0.33 ± 2.6	+0.08 ± 3.2	-0.87 ± 2.0	0.22	0.17	0.29
Total	*Baseline*	71.5 ± 25	73.8 ± 31	64.1 ± 32	74.9 ± 29			
	Month 6	65.9 ± 27	66.2 ± 25	68.8 ± 25	72.8 ± 28			
	Δ	-6.5 ± 22	-8.5 ± 29	+1.1 ± 41	-2.8 ± 38	0.44	0.16	0.87

[A]Sum of isobutyric acid, isovaleric acid and 2-methyl-butyric acid

[B]Non-parametric analyses

Supplemental Materials and Methods

qPCR analysis for *Bifidobacterium animalis* ssp. *lactis* 420 in feces

Fecal samples were stored at -80℃ before analysis. DNA from each sample was extracted and purified with a commercial kit (MagMAX™ Total Nucleic Acid Isolation Kit, Applied Biosystems, Bridgewater, NJ, USA) followed by PCR inhibitor removal in 96-well format silica columns (OneStep-96™ PCR Inhibitor Removal Kit, Zymo Research, Irvine, CA, USA). DNA concentrations were determined by Qubit™ 2.0 Fluorometer using Qubit® dsDNA HS Assay Kits (Life Technologies, Darmstadt, Germany). 1 ng of sample DNA was used in each PCR reaction. A qPCR assay targeting *Bifidobacterium animalis* ssp. *lactis strains:* B420 was used; the primers also detect strains HN019, Bl04 and Bi07 (primers shown in table). Primers were designed using Geneious (Biomatters Ltd., Auckland, NZ) and Mauve multiple genome alignment tool (http://darlinglab.org/mauve/mauve.html). Specificity was verified *in silico* with NCBI BLAST (http://blast.ncbi.nlm.nih.gov/Blast.cgi). The amplification, detection and melt curve analysis of DNA were performed on an ABI-PRISM FAST 7500 sequencing detection system using included 7500 Software v2.0.6 (Applied Biosystems). The reaction conditions were: 20s at 95℃, 40 cycles of: 3s at 95℃; 30s at 58℃ followed by disassociation curve cycling. A standard curve from 10 ng and 100 fg of genomic DNA extracted from a pure strain culture was used. No values detected below lowest standard or those whose melt curves did not match that of the standard, were accepted. Each plate was run with non-template control. The samples at 6 months were compared to baseline samples, and participants were considered positive for compliance when the B420 concentration was at least one log higher at the 6-month visit compared to baseline. Participants were considered test negative when the B420 concentration was undetectable at 6 months or when the concentration remained within one log compared to baseline.

Primer Name	Sequence 5'-3'	Tm (°)	Target
Blac_glcU_fwd	CCTGCATGGGGAATCGTGTA	58.9	glucose uptake permease
Blac_glcU_rev	TCCTAGAATGCTTGTGTTTTGCG	57.3	glucose uptake permease

Analysis of exploratory laboratory outcomes

Plasma and serum were aliquoted and stored at -80℃ until analysis. Blood biomarkers were determined by using multiplex ELISA panels (Aushon Biosystems, Billerica, MA) with intra- and inter-assay CV%s as follows: interleukin-6 (IL-6) (7%, 6%), tumor necrosis factor alpha (TNF-α) (3%, 7%), IL-1β (5%, 6%), plasminogen activator inhibitor 1 (PAI-1) (9%, 4%), vascular cell adhesion protein 1 (VCAM-1) (4%, 3%), intracellular adhesion molecule 1 (ICAM-1) (4%, 3%), E-selectin (4%, 2%), soluble cluster of differentiation 14 (sCD14) (4%, 5%), leptin (7%, 5%), monocyte chemoattractant protein-1 (MCP-1) (11%, 2%) and adiponectin (3%, 12%). In addition, we used commercially available ELISA kits for zonulin (Immunodiagnostik, K5601, intra-assay CV 4.5%, inter-assay CV 9.3%), angiopoietin-like protein 4 (angptl4) (Biovendor, RD191073200R, intra-assay CV 4.3%, inter-assay CV 6.12-10.69%), oxidized LDL cholesterol (oxLDL) (Mercodia, 10-1143-01, intra-assay CV 3.3%, inter-assay CV 6.8%), and ApoB-48 (Shibayagi, AKHB48, intra-assay CV 4.2%, inter-assay CV 12.2%).

Analysis of bacterial fermentation products in feces

Fecal samples were stored at -80℃ until they were thawed on ice for analysis of short-chain fatty acids (SCFA) with gas chromatography, as described previously (Ouwehand et al. 2009). An internal standard (0.1 ml 20 mM pivalic acid) and 0.5 ml of water were added to 0.1 g of sample. After thorough mixing, the sample was centrifuged at 5000 x g for 5 min. Saturated oxalic acid solution (250 µl) was then added to 500 µl of the supernatant and the mixture was incubated at 4°C for 60 min, and centrifuged at 16000 x g for 5 min. The supernatant was analyzed by gas chromatography using a glass column packed with 80/120 Carbopack B-DA/4% Carbowax 20 M stationary phase (2 m x 2 mm, Supelco, Bellefonte PA, USA) at 175°C and using

helium as the carrier gas at a flow rate of 24 ml/min. The temperatures of the injector and the flame ionization detector were 200°C and 245°C, respectively. The limits of determination were 0.1 µmol/g for acetic acid, propionic acid, butyric acid, isobutyric acid, valeric acid, isovaleric acid and 2-methylbutyric acid, and 1 µmol/g for lactic acid.

Measurement of food intake

The participants were instructed to fill in a 5-day food diary prior to the baseline, 2-month and end-of-intervention clinic visits. The participants used a portion size booklet (Lehtisalo et al., 2013) to improve accuracy. Qualified nutritionists analyzed the food diary data with AivoDiet software (Aivo Finland Oy, Finland) using a national database of food ingredients and their composition (Fineli, National Institute of Health and Welfare, Finland). The energy content of the smoothie vehicle was included in the energy intake data of visits during the intervention. A basal metabolic rate was calculated for each subject according to their baseline body weight, height and age. Food diaries with energy intake below 80% of the basal metabolic rate for women and 85% for men were regarded as underreported and consequently excluded from the analyses. These food diaries accounted for 1-2 participants per group, evenly from each group.

Supplemental references

Lehtisalo, J., Lindström, J., Nieminen, R., and Paturi, M. (2013). Annoskuvakirja - Ruokapäiväkirjan täyttämisen tueksi. (Helsinki:The Finnish National Institute of Health).

Ouwehand, A.C., Tiihonen, K., Saarinen, M., Putaala, H., and Rautonen, N. (2009). Influence of a combination of Lactobacillus acidophilus NCFM and lactitol on healthy elderly: intestinal and immune parameters. The British journal of nutrition *101*, 367-375.
